# Land-use change in oil palm dominated tropical landscapes—An agent-based model to explore ecological and socio-economic trade-offs

**DOI:** 10.1371/journal.pone.0190506

**Published:** 2018-01-19

**Authors:** Claudia Dislich, Elisabeth Hettig, Jan Salecker, Johannes Heinonen, Jann Lay, Katrin M. Meyer, Kerstin Wiegand, Suria Tarigan

**Affiliations:** 1 Ecosystem Modelling, Faculty of Forest Sciences and Forest Ecology, University of Goettingen, Goettingen, Germany; 2 Faculty of Economic Sciences, University of Goettingen, Goettingen, Germany; 3 GIGA German Institute of Global and Area Studies, Hamburg, Germany; 4 Centre of Biodiversity and Sustainable Land Use (CBL), University of Goettingen, Goettingen, Germany; 5 Soil and Natural Resources Management, Bogor Agricultural University, Bogor, Indonesia; National University of Singapore, SINGAPORE

## Abstract

Land-use changes have dramatically transformed tropical landscapes. We describe an ecological-economic land-use change model as an integrated, exploratory tool used to analyze how tropical land-use change affects ecological and socio-economic functions. The model analysis seeks to determine what kind of landscape mosaic can improve the ensemble of ecosystem functioning, biodiversity, and economic benefit based on the synergies and trade-offs that we have to account for. More specifically, (1) how do specific ecosystem functions, such as carbon storage, and economic functions, such as household consumption, relate to each other? (2) How do external factors, such as the output prices of crops, affect these relationships? (3) How do these relationships change when production inefficiency differs between smallholder farmers and learning is incorporated? We initialize the ecological-economic model with artificially generated land-use maps parameterized to our study region. The economic sub-model simulates smallholder land-use management decisions based on a profit maximization assumption. Each household determines factor inputs for all household fields and decides on land-use change based on available wealth. The ecological sub-model includes a simple account of carbon sequestration in above-ground and below-ground vegetation. We demonstrate model capabilities with results on household consumption and carbon sequestration from different output price and farming efficiency scenarios. The overall results reveal complex interactions between the economic and ecological spheres. For instance, model scenarios with heterogeneous crop-specific household productivity reveal a comparatively high inertia of land-use change. Our model analysis even shows such an increased temporal stability in landscape composition and carbon stocks of the agricultural area under dynamic price trends. These findings underline the utility of ecological-economic models, such as ours, to act as exploratory tools which can advance our understanding of the mechanisms underlying the trade-offs and synergies of ecological and economic functions in tropical landscapes.

## Introduction

Land-use changes have dramatically transformed tropical landscapes throughout the past decades. Large stretches of pristine rainforests, grasslands, and peatlands have been replaced by agriculture [1]. This process continues, and even transformed landscapes are subject to continued human-induced land-use changes. Traditional smallholder agricultural systems are particularly turned into intensified monoculture cash crop plantations, for example, oil palm or rubber plantations [2]. It is well-documented that the replacement of previous forests, grasslands, traditional agricultural systems, or fallow lands by intensified agricultural systems can lead to losses in ecosystem functions [3–5]. At the same time, agricultural intensification provides opportunities for economic development, especially in poor rural areas in developing countries [6, 7].

Interdependencies of ecosystem functions are present both within and between the ecological and socio-economic spheres. For example, the carbon storage ability of a landscape depends on ecological properties such as soil fertility, but may additionally be altered by human interaction and agricultural land use. Such interactions are likely to be complex, often non-linear, and not well-understood.

This paper presents an agent-based simulation model to analyze the ecological and economic drivers and consequences of land-use change in transformed landscapes in the Jambi Region of Sumatra, Indonesia. In these landscapes, oil palm and rubber represent the dominant land-use types. Our study focuses on how the decisions of smallholder famers with typical field sizes of around two hectares shape the landscape mosaic and the corresponding economic and ecological functions. The model relies on detailed socio-economic and ecological data collected with an integrated design and provided by the interdisciplinary research project EFForTS (Ecological and Socioeconomic Functions of Tropical Lowland Rainforest Transformation Systems, Indonesia), which started in 2012 (see [8]). We therefore refer to our model as EFForTS-ABM (Agent-Based Model).

Drawing on the comprehensive EFForTS database, our guiding research question is: What kind of landscape mosaic can improve the ensemble of ecosystem functioning, biodiversity, and economic benefit based on the synergies and trade-offs that we have to account for (cf., [9])? This leads to more detailed questions: (1) How do specific ecosystem functions, such as carbon storage, and economic functions, such as household consumption, relate to each other? (2) How do external factors, such as the output prices of crops, affect these relationships? (3) How do these relationships change when production inefficiency differs between smallholder farmers and learning is incorporated?

Agent-based ecological-economic simulation models are suited to answer these questions [10]. Agent-based simulation models (ABMs) are considered as “*across-level* models” [11, p. 10]; that is, they can simulate the behavior of the system from that of individual agents and vice versa. They can thus incorporate the individual decisions of agents, for example, farming households, and evaluate the effects of these decisions on ecological and socio-economic functions at different scales (for example, local or landscape scales). These individual household decisions are themselves driven by social (adaption, learning) and environmental interactions (resources, yield). Such capacities of the often spatially explicit ABM approach have been used to tackle a variety of real-world situations [12–14] and many of the models are based on empirical data to the extent possible. Our model adds to this literature by highlighting the key role of smallholder behavior in the ecological-economic system under consideration. More specifically, we take into account the full extent of household farm production inefficiency and model social network learning processes based on spatial proximity (cf. [15–17]). Our modelling of household behavior is based on a dataset of 701 farm households with information on land holdings, agricultural and non-agricultural activity, endowments, and other socio-demographic information [18, 19]. High heterogeneity and high average yield gaps in cash crops are well-documented for our study region (for example, in oil palm, see [20]). On the ecological side, we focus on carbon storage, again using original data from EFForTS. Considering other ecosystem functions, ongoing research will provide land-use-specific data covering the whole spectrum of ecological functions, such as species composition, or water availability and water quality.

EFForTS-ABM is complex enough to capture all factors and processes relevant to our questions (1)-(3), yet simple enough to make the model mechanisms and the forces that drive outcomes tractable (cf., [21]). We analyze the relationships between economic and ecological functions using a scenario-based approach. We highlight land-use decisions, changes in the landscape and the corresponding functions (household consumption and carbon sequestration) under (a) different output price scenarios, (b) different farming efficiency scenarios, and (c) different assumptions on learning. This approach enables us to segregate the different mechanisms that drive ecological-economic trade-offs, such as price dynamics and household heterogeneity. EFForTS-ABM thereby enables us to gain a deeper understanding of the dynamic agricultural smallholder land-use system. This is an essential step for future research that will allow for the testing and devising of specific land-use and agricultural policies aimed at the improvement of the ecosystem functioning of transformed tropical landscapes.

## Methods

The model description is structured according to the ODD+D protocol [22], an extension to the ODD protocol for describing agent-based models [23, 24] which incorporates human decision-making.

### Overview

#### Purpose

The purpose of our model is to provide an integrated, exploratory tool that can be used to analyze how land use affects ecological and socio-economic functions. Trade-offs or synergies between different functions on different spatial and functional scales will be investigated with the model.

As smallholders manage the majority of farm land in our study region, we focus on smallholder land management at this point. However, large company plantations will be added in future extensions of the model (see Section Outlook). Land-use and land-management decisions are modelled on the household level, based on household capital and external economic drivers like prices for inputs and products. Socio-economic functions in the model are development and welfare effects; on the ecological side, we focus on carbon storage. Further ecological functions, for example, species diversity, will be incorporated in the near future. We consider the perennial land-use types oil palm and rubber plantations, and use secondary forest (as a near-natural habitat) as a background in the landscape matrix, but we do not analyze secondary forest explicitly for the purposes of this paper.

We choose a spatially explicit approach by discretizing space into grid cells, since the location of the household and its farmland in the landscape might affect the decision-making process, as well as ecological functions. For example, the spatial distance between households influences whether households can learn from each other. Further, biodiversity can be affected by the degree of landscape fragmentation. A combined agent-based and grid-based approach provides the flexibility needed to model diverse ecological and socio-economic functions. Interactions between grid cells, as well as interactions between households, can be included explicitly in such a framework.

#### Entities, scales, and state variables

The model simulates ecological and socio-economic aspects of land use and land-use change and therefore comprises five different entities: (1) cells, (2) fields, (3) household area, (4) patches, and (5) the landscape (see Table 1). These spatial units capture the hierarchical structure of the system and facilitate the structurally realistic representation of the links between ecology (environment) and socio-economics (households).

**Table 1:** Spatial units of the model.

Spatial unit	Meaning
cell	smallest spatial unit of the model (50 *m* × 50 *m*)
field	contiguous cells of the same land-use type and age belonging to the same household (that is, an agricultural field)
household area	cells belonging to the same household
patch	contiguous cells of the same land-use type and same/similar age (that is, same type of habitat, independent of ownership)
landscape	largest spatial unit of the model: set of all cells

**Fig 1:**
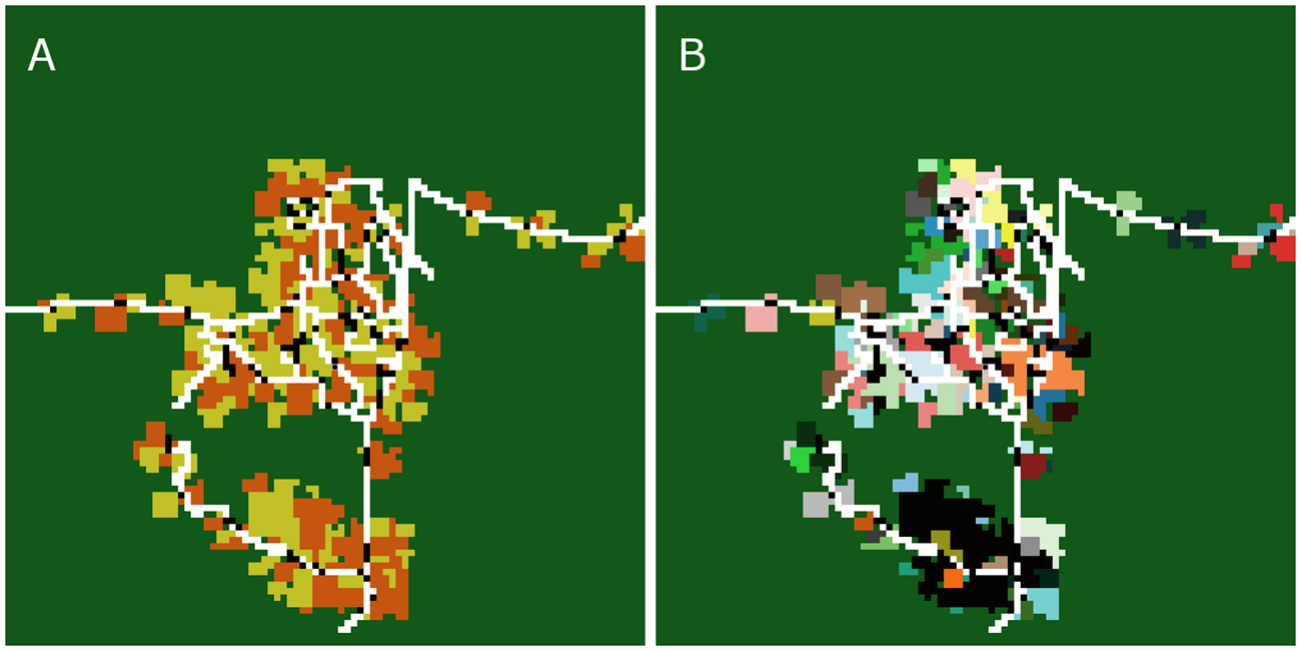
(A) Initial land-use map. Roads are marked in white, household home bases in black, oil palm plantations in orange, rubber plantations in dark yellow. Dark green is the area which is not used for agriculture. (B) Household map: different colors represent areas of different households.

The smallest spatial unit of the model is a square cell corresponding to the typical size of small fields (50 *m* × 50 *m*; Fig 1(A)). Each cell is characterized by its position in the landscape, land-use type, and age, which is the time (number of years) since the current land-use was established. A field is defined as a number of contiguous cells under the same use for the same duration belonging to one household. Each household can own several fields and decide on their use and management (Fig 1(B)). We do not consider expansion of household area or agricultural land and the size of existing fields remains constant throughout each simulation. Similar to fields, patches are contiguous cells of the same use and the same (or similar) age, but regardless of ownership. While fields are important units in the economic sub-model, patches define areas of similar habitat suitability and may thereby play an important ecological role for species diversity and distribution. The landscape comprises a regular grid of cells and is the highest-level entity of the model (here 100 × 100 cells, that is, 25 *km*^2^). All processes in the model, for example, vegetation growth, as well as household-related processes, work on an annual time step. Prices for yield are external and do not vary within the landscape. The temporal extent of one simulation is 50 years according to the external price data that was provided (historical prices scenario; see Section *Price dynamics, Appendix A in* S1 File).

Households are characterized by their location in the landscape, the sizes and locations of fields belonging to the household, and specific characteristics, such as wealth. Household variables describe the size and production of the owned land, as well as the financial resources of the household (details in Table 2). A detailed description of the household model is given in *Appendix A in* S1 File.

**Table 2:** List of household variables.

Variable name	Unit	Meaning
h_id	[-]	Household identifier
h_area	[-]	Number of cells belonging to the household
h_wealth	[USD]	Amount available for the household
h_inefficiency_op	[-]	Inefficiency factor for oil palm [0, 1]
h_inefficiency_rubber	[-]	Inefficiency factor for rubber [0, 1]
h_debts	[USD]	Annual debts taken up for agricultural production
h_capitalstock	[USD]	Amount of capital fixed in plantations
h_exincome	[USD]	Annual external income, i.e. income external to agriculture
h_netcashflow	[USD]	Net cash flow from all household cells
h_consumption	[USD]	Annual consumption of household (fixed + variable consumption)
h_cost_investment	[USD]	Annual investment costs from all household cells
h_cost_labor	[USD]	Annual labor costs from all household cells
h_cost_tinput	[USD]	Annual technical input costs from all household cells
h_cost_capital	[USD]	Annual capital costs from all household cells
h_cost_land	[USD]	Annual land rent costs from all household cells
h_revenue	[USD]	Annual revenue from agriculture
h_op_production	[ton]	Annual production of oil palm fruit bunches from all household cells
h_rubber_production	[ton]	Annual production of rubber from all household cells
h_debt_years	[-]	Number of consecutive years in which the household had debts > 0
h_connected_hhs	[-]	h_ids of connected households, representing the social network

Cell variables describe ecological and economic properties of the land use in that respective cell, such as type of use (for example, oil palm), age, technical input, production, and amount of carbon stored in the vegetation of that cell (details in Table 3).

**Table 3:** List of cell variables.

Variable name	Unit	Meaning
p_landuse	[-]	Land use of the cell (oil palm, rubber, secondary forest)
p_age	[year]	Age of the plantation in the cell
p_fieldsize	[-]	Total number of cells belonging to the same field as this cell
p_carbon	[ton]	Carbon stored in the vegetation of this cell
p_owner	[-]	h_id, if this cell is owned by a household, otherwise −1
p_homebase	[-]	h_id, if this cell is the homebase of a household, otherwise −1
p_production	[ton]	Annual production from this cell
p_id	[-]	Field identity; all cells belonging to the same field have the same field identity
p_labor	[h]	Labor hours invested in this cell in one year
p_tinput	[kg]	Technical input invested in this cell in one year
p_capitalstock	[USD]	Capital stock of this cell

#### Process overview and scheduling

**Fig 2:**
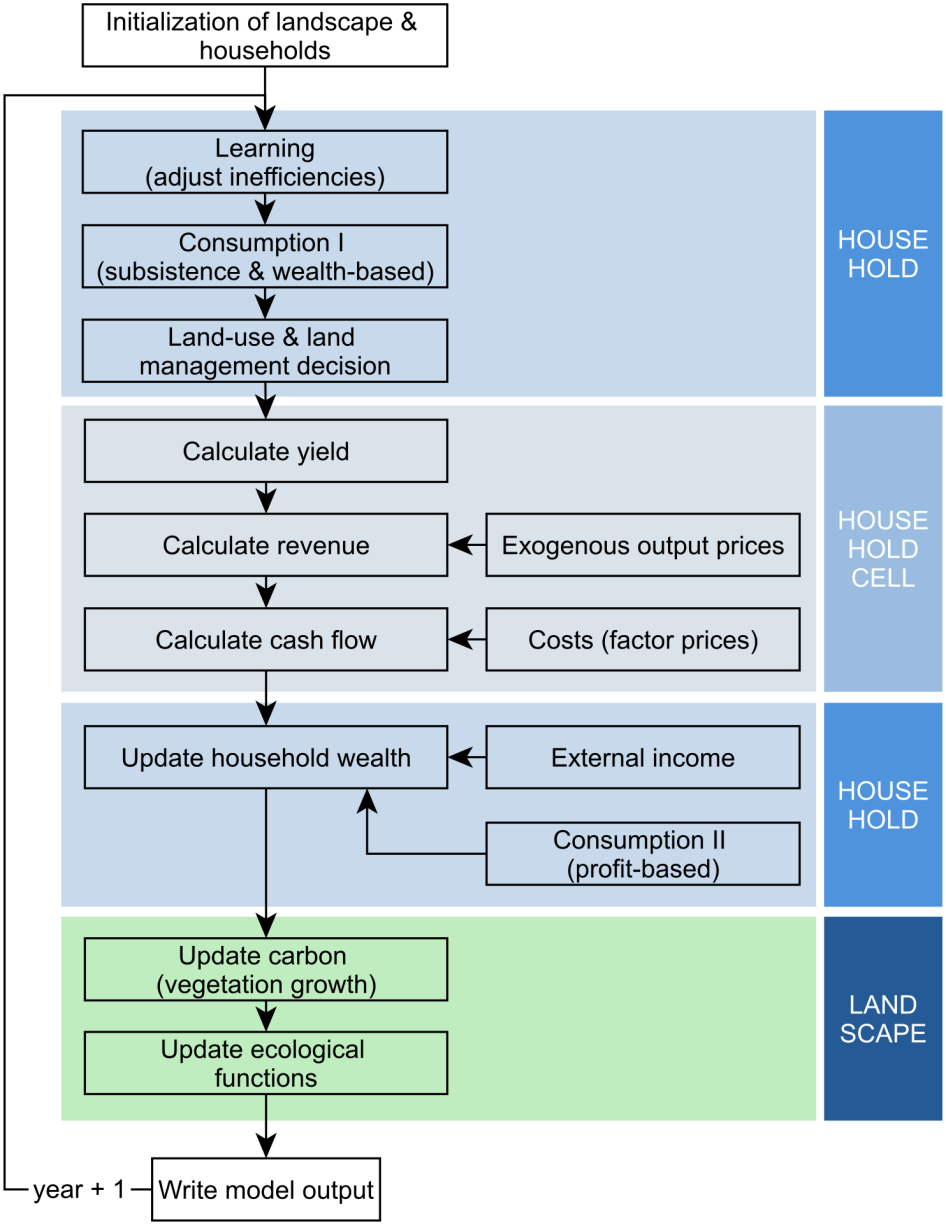
Process overview of the whole model.

Each model run starts with the initialization procedure, with the loading of a pre-generated initial landscape that is parameterized to our study region and features household agents that are located on a road network, as well as agricultural fields which the agents have established (see *Appendix B in* S1 File). During initialization, crop-specific inefficiency values are also assigned to these households (see Section *Initial household inefficiency distribution, Appendix A in* S1 File). After initialization, each grid cell has a certain land use (oil palm, rubber, or secondary forest). Each grid cell under agriculture (oil palm or rubber) has an owner and a certain age. Each household has a predefined initial wealth which is proportional to the area belonging to the household (see Section *Initialization, Appendix A in* S1 File and *Initial household wealth, Appendix C in* S1 File). Within each time step (year), the following processes are scheduled (Fig 2). At the beginning of each year, the economic household model is executed. If the optional learning is turned on, households adjust their inefficiency values by learning from their social network (see Section *Household inefficiency & learning, Appendix A in* S1 File). Household consumption, which is subsistence and wealth-based, reduces the available resources for agriculture (Consumption I, for details, see Section *Decision on land-use change and production, Appendix A in* S1 File).

**Fig 3:**
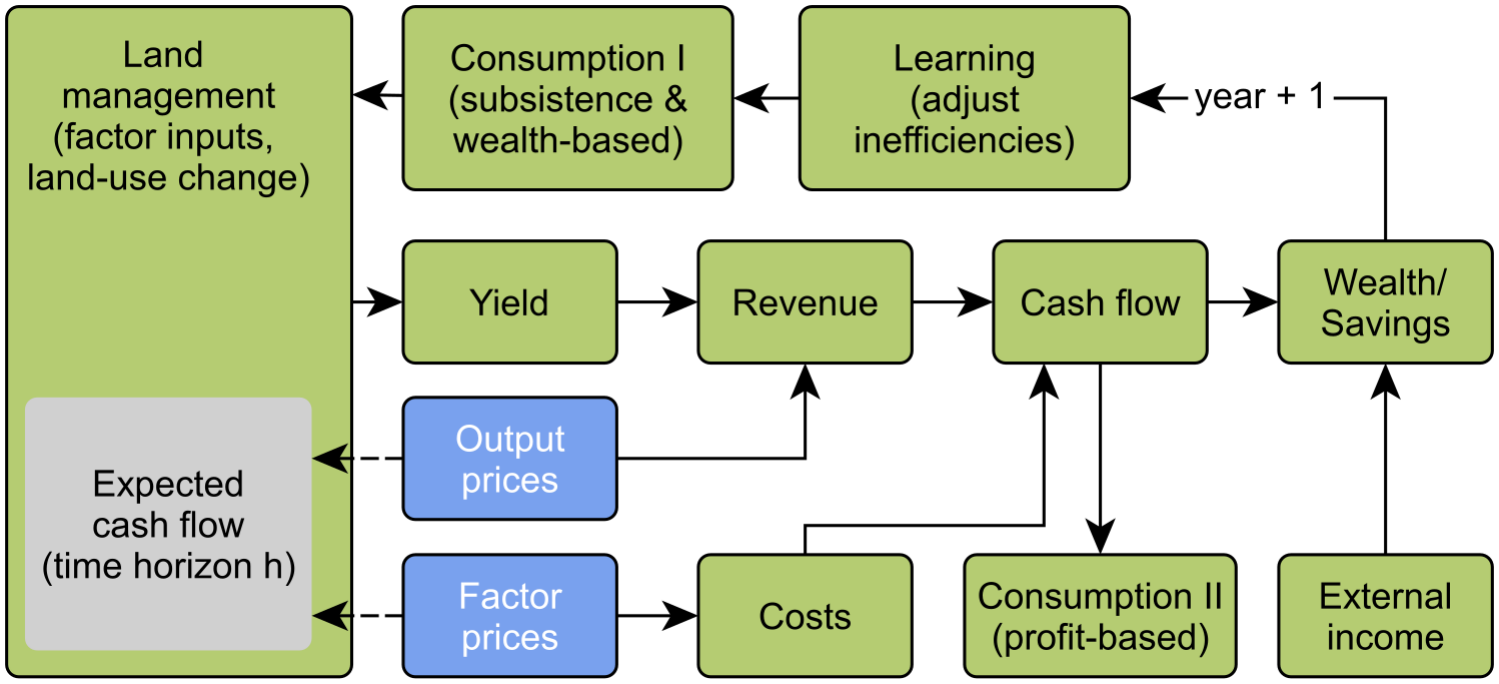
Process overview of the economic household model.

Subsequently, households decide on land management and land-use change (Fig 3). This decision is based on expected profits from different land-use options and available financial resources. The actual annual profit from agricultural land use is then calculated for all household cells according to age-specific yields and costs and prevailing commodity prices. At this point, the costs of land-use change, if applicable, are accounted for. These costs for land management include crop and age-specific maintenance and establishment costs (see Section *Optimal capital input, Appendix A in* S1 File). Household wealth is then updated by adding profits from agriculture and external income (if applicable) and deducting a profit-based household consumption (Consumption II). Households may temporarily take up debts to cover consumption or unavoidable costs (see Section *Implementation of the land management decision, Appendix A in* S1 File). Households that do not manage to pay back debts within a certain period are assumed to be incapable of acting and become frozen in the model. Home bases and fields of frozen households disappear from the visual output and are not considered for any upcoming output calculations.

The updated household wealth serves as a basis for the land-use decision module in the next time step. When the calculation of the economic household model is finished, new carbon stocks are calculated for all cells.

### Design concepts

#### Theoretical and empirical background

**Economic household model** The economic household model is based on the concept of “agricultural household models” [25]. In this type of model, a rural household simultaneously decides on production and consumption under given constraints, for example, initial endowments with land or access to credit. The land management decision includes land-use change and production, as well as the use of factor inputs.

**Ecological sub-models** The currently applied carbon sub-model describes carbon stored in the vegetation and utilizes simple age-dependent stock equations for the oil palm and rubber plantation land-use types, and constant stock values for forest cells. Other factors that might influence carbon stocks, for example, edaphic conditions, fertilizer management, etc., are not considered in this model version.

#### Individual decision-making

Every year, households decide on the land management and land-use change of owned fields. These decisions are driven by their agricultural choices, which, in turn, are determined by production technologies, initial conditions, and household endowments. Households attempt to maximize profits and decide between different land uses according to the expected relative profitability of different options over a certain time horizon. Constraints are taken into account, for example, with regard to the availability of investment capital via proceeds from agricultural production.

#### Individual learning, sensing, and prediction

When learning is turned off, each agent makes its decision independently; that is no neighbor effects are incorporated. When learning is turned on, agents are able to learn from more efficient ones within their individual social network. These social networks are created during model initialization, based on the spatial proximity of agents using road distances. Households are only able to improve the inefficiency of a crop they currently cultivate (“learning by doing”) and if their inefficiency is above the current network’s mean inefficiency level. By learning, households continuously adjust their inefficiency values to the network’s mean inefficiency level (see Section *Household inefficiency & learning, Appendix A in* S1 File).

Agents’ knowledge of prices is restricted to current commodities. Therefore, they forecast future prices using current prices and anticipate zero change. This is also true for inefficiencies; agents do not anticipate their own learning.

#### Interaction, collectives, and heterogeneity

When learning is turned on, the model incorporates interactions between agents. Irrespective of learning, agents differ in their land and capital endowments, as well as their initial land uses and the ages of their fields. Additional factors that introduce heterogeneity are the inefficiency parameters (one for each crop type; see Table 2) that affect a household´s production function (see Section *Household model, Appendix A in* S1 File).

#### Stochasticity

The initial wealth of households is drawn from a log-normal distribution and resulting values are assigned according to household areas (see Section *Initialization, Appendix A in* S1 File, and *Appendix C in* S1 File). Parameters for crop and household-specific inefficiency are drawn from gamma distributions and either stay constant or vary through learning. Both wealth and inefficiency parameters are drawn from distributions using observed data. The social networks used by the learning procedure can be created either deterministically or stochastically by using a negative exponential probability function (for details on initialization, see *Appendix A in* S1 File).

Different options of stochastic price dynamics are implemented (for example, Gaussian random walk; see Section *Price dynamics, Appendix A in* S1 File). However, this option can be turned off to use constant prices.

#### Observation

Observed household patterns include land-use changes and dynamic yield development, predicted and actual cash flows, and household wealth. On the landscape level, we observe the fractions of different land-use types, mean consumption, survival of households, and carbon stocks.

### Model scenarios

In order to address our research questions (1)-(3), and to explore underlying model mechanisms, we run six different model scenarios (see Table 4). We analyze the spatial patterns and economic and ecological trade-offs that emerged from the land-use decisions of households. We look at two different price scenarios: (i) constant prices for oil palm and rubber, set to values derived from the household survey data, collected in the Jambi region in 2012; (ii) historical price trends, which illustrate actual price fluctuations in world commodity markets, derived from World Bank data (see Section *Price dynamics, Appendix A in* S1 File). For each price scenario, we look at three different household inefficiency scenarios: (i) all farmers have zero inefficiency for both crops (that is, all are perfectly efficient); (ii) farmers have heterogeneous inefficiency values drawn from gamma distributions. Inefficiencies stay constant throughout the simulation (that is, learning is turned off); (iii) farmers have heterogeneous inefficiency values and are allowed to improve productivity through learning from their social network (that is, learning is turned on). The social networks are created by using the distance kernel function combined with a maximum distance cap (see Section *Household inefficiency & learning, Appendix A in* S1 File). The temporal extent of all scenarios is 50 years, based on the extent of the external price data. In order to capture model stochasticity we simulate 20 replications for each scenario.

**Table 4:** Model scenarios.

Scenario	Prices	Inefficiency
C0	Constant prices (C)	No inefficiencies (0)
CI	Constant prices (C)	Heterogeneous inefficient households and no learning (I)
CIL	Constant prices (C)	Heterogeneous inefficient households and learning (IL)
H0	Historical trends (H)	No inefficiencies (0)
HI	Historical trends (H)	Heterogeneous inefficient households and no learning (I)
HIL	Historical trends (H)	Heterogeneous inefficient households and learning (IL)

## Results and discussion

The main result of this study is an integrated ecological-socio-economic land-use model, called EFForTS-ABM. EFForTS-ABM is an integrated model in terms of disciplines, processes, and scales (for spatial scales, see Table 1). Thereby, it covers key features of integration in environmental modelling [26].

The purpose of the model is to develop system understanding rather than prediction or forecasting [26]. We chose an agent-based modelling approach because it incorporates complexity and details at the individual level [26], including heterogeneity between agents, as well as interactions between agents and scaling up from the agent to the landscape level [27]. We have both quantitative and qualitative data available on social, economic, and ecological functions of the system [28, 29]. Both the qualitative purpose and the agent-based approach of the model facilitate incorporating different types of data. Moreover, we are in the unique situation that data collection follows an integrated scheme that has jointly been developed by modelers and empiricists [8, 30, 31]. Thus, the relationship between the data collection and monitoring in the field and the modelling can resemble an integrated environmental process, with feedbacks between different stages of the two procedures, providing a more holistic approach [32].

Our approach is distinct from previous agent-based models of land-use change that have been developed for Jambi, Sumatra, (LB-LUDAS [10]) and for other study regions and other land uses (e.g. [33, 34]). This holds in particular for the decision-making process of the smallholders. We concentrate on (i) an appropriate representation of the intertemporal nature of the land-use change decisions implied by investment costs (related to switching crops or replanting) and the respective yield cycles, and (ii) an incorporation of the heterogeneity of smallholders in key determinants of land-use change decisions, most notably their endowments with land, capital, and labor, as well as crop-specific productivity [35]. We especially consider the incorporation of productivity heterogeneity, i.e. in production parameters, which is an innovation of our study. Further, we (iii) let farm households decide simultaneously on production and consumption, as is well-established in the literature on decision-making in agricultural households [36]. Data from a smallholder survey of relatively large size (701 farm households) allows us to quantify all these household and farm-level variables [18, 19]. We follow previous models (for example, [34]) and the literature on agricultural household models by assuming a rational “portfolio” choice that is constrained by household endowments and, in fact, subsistence needs. Note that the context that we are modeling is characterized by smallholder cash-croppers who are relatively undiversified in terms of agricultural production. All these modeling choices keep our model and the effects of smallholder heterogeneity tractable. We acknowledge, however, that this simplicity puts limitations to our analysis. The representation of the smallholder decision-making and their heterogeneity emphasizes the relative profitability of the two cash crops as a key driver of land-use change. The model does not explicitly account for socio-demographic household characteristics, such as education or ethnicity. Another important omission is the non-consideration of risk and risk aversion, as in the heterogeneous-agent portfolio-theory model by Kelley and Evans [34]. This limitation may not be too severe as the risks related to the two considered crop options may be similar. Thus, our model has less probabilistic elements than, for example, Valbuena et al. [33]. While we do not explicitly model differences in preferences (as LB-LUDAS [10]), the fact that the crop-specific productivity at the farm-level is parameterized in line with observed crop choices can be seen as an equivalent model feature.

Unlike previous applications of ABMs (e.g. [33, 34]), the focus of EFForTS-ABM is on the human-environment interactions of transformed landscapes. It is thus not on the expansion of agricultural land because most of the forested area in our study region has already been converted to monoculture plantations over the past decades. In contrast to other applications, we therefore do not consider land expansion. In addition, land-use options have been more differentiated in other models. Kelley and Evans [34], for example, include fallow land as an option, as farmers might want to reduce labor costs if output prices are low. In the EFForTS-ABM model, neighborhood effects work through learning effects, which indirectly influence land-use change decisions. Admittedly, direct neighborhood effects, i.e. where land conversion by one farmer directly affects the conversion decision of the neighbor, may be an important mechanism that EFForTS-ABM does not yet capture—this is also because we lack data on such conversion patterns. Further, other spatial aspects, including the composition of the surrounding landscape and transaction costs, such as the distance to roads (or processing facilities), are not yet accounted for.

**Fig 4:**
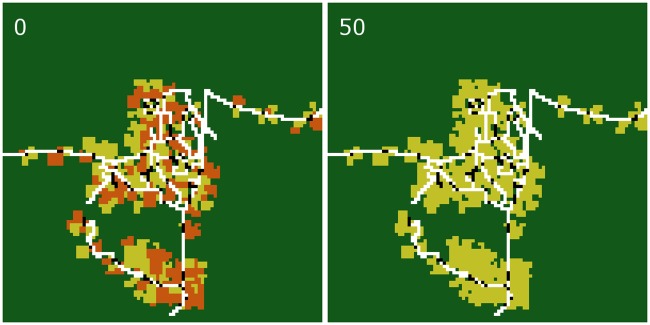
Snapshots of the initial (year 0) and final (year 50) simulated landscape of an exemplary simulation run with constant prices and no inefficiencies (scenario C0). Roads are marked in white, household home bases in black, oil palm plantations in orange, and rubber plantations in dark yellow. Dark green is the area which is not used for agriculture. An animated representation of this example simulation can be found in Appendix S1 Fig: *Default simulation run animation*.

**Fig 5:**
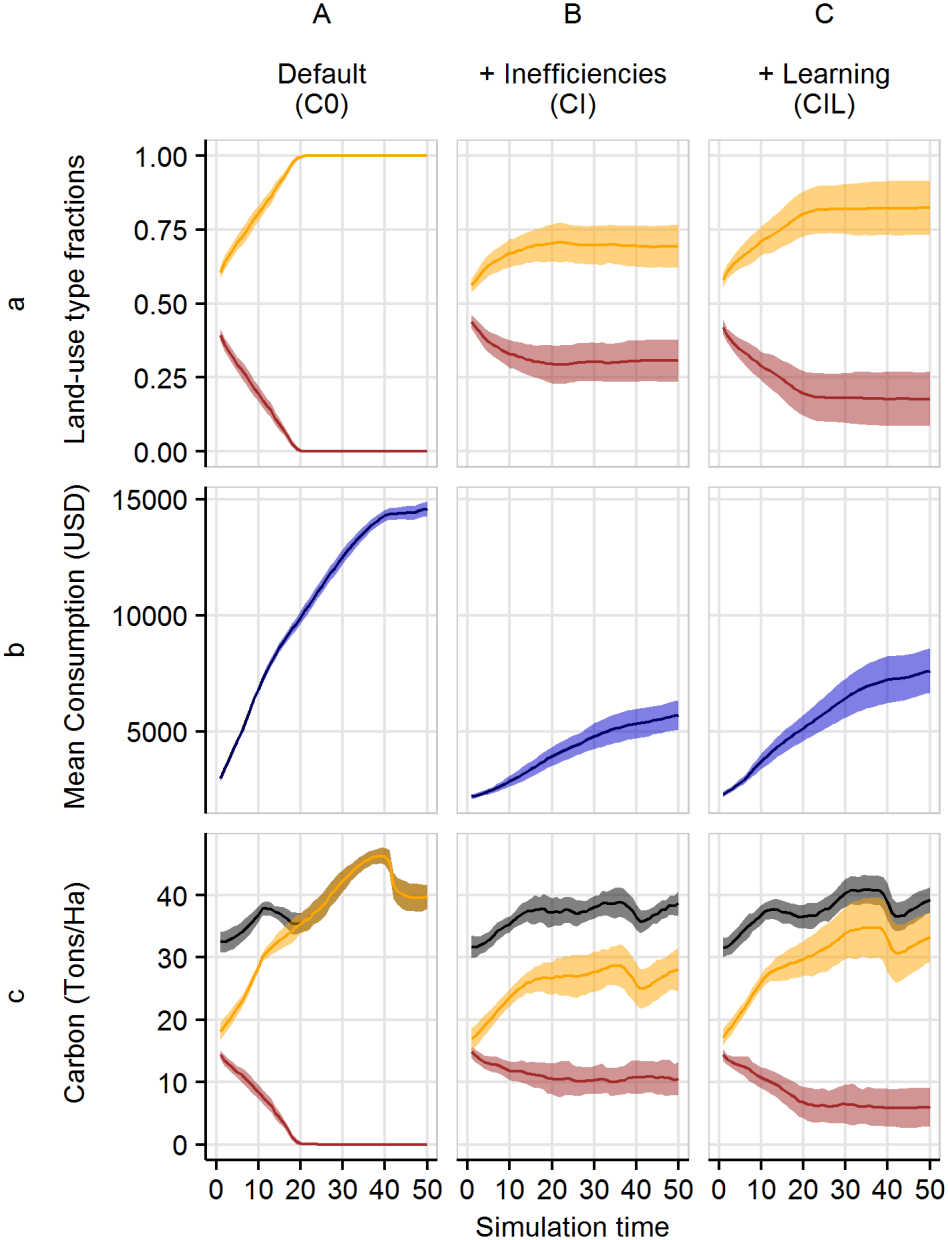
Temporal dynamics under constant price scenarios. Fractions of different land-use types within the agricultural area (a), mean household consumption (b), and vegetation carbon stocks over time (c), with constant output prices; for agents with no inefficiencies and no learning (A), heterogeneous inefficiencies and no learning (B), and heterogeneous inefficiencies and learning (C). In (a) and (c), the light orange color denotes rubber monoculture, while red denotes oil palm. In (c), black refers to the total carbon of land under agriculture. The dark lines represent mean values of the 20 model replications and the shaded polygon surrounding each line represents the respective standard deviations.

The key mechanism of the EFForTS-ABM in its current form is the household´s land management decision. Farmers will tend towards the more profitable land use and will convert land with some time lag conditional on the current land use. For instance, the household’s capital endowment needs to be sufficient enough to cover the investment costs of conversion. This implies that the model should produce convergence towards the more profitable land use, at least if productivity is homogeneous and input and output prices are constant and common to all farmers. Indeed, we observe this behavior in the model. For example, at the farm-gate prices of the last quarter of 2012 with rubber at US$1,100 per ton and oil palm at US$90 per ton of fresh fruit bunches (FFB), rubber turns out to be more profitable than oil palm regardless of the time horizon used for how far into the future the household calculates expected net cash flows (time horizon tested up to 20 years, see Section *Costs, revenue & Cash flow, Appendix A in* S1 File). In such a scenario and with default settings, the proportion of fields planted with rubber increases to 1.0 (Figs 4 and 5(A, a)). The transition phase from a proportion around 0.5 for both crops in the initial situation to a complete dominance of rubber is about 20 years under the current model specification and parameterization.

The model produces more diverse land-use patterns if we introduce heterogeneity in productivity, that is, differences in household inefficiencies (Fig 5(B, a)). In this case, the relative profitability of rubber and oil palm differs between households and therefore also their choice. For some households, oil palm is more profitable than rubber, despite low output prices; this is due to these households having a substantially higher inefficiency for rubber than for oil palm. When learning is added, land-use change patterns are still inert, but they move closer to the scenario without inefficiencies (Fig 5(C, a)). Learning enables farmers to improve productivity over time and they become more homogeneous.

The simulated land-use change scenarios under constant prices are associated with considerable increases in household consumption (Fig 5(b)). In general, and in the absence of learning, two forces are at work in the model that can increase profits and thus consumption over time. One is the “natural” yield growth of both crops over time (see Section *Production functions for oil palm and rubber, Appendix A in* S1 File); the second force is the option to switch to a more profitable crop. However, the investment costs of switching will cut into consumption and may temporarily decrease household welfare. The model results show the average implications of these consumption-level mechanisms (Fig 5(A, b)). Overall, consumption more than doubles within about 20 years. This is driven by both switching to more profitable rubber, as well as increasing yields with plantation age. An increase in yields clearly drives the observed consumption increase after year 15, because most of the households already switched all their fields to rubber. After year 40, the growth of consumption slows down again as the necessary replanting of rubber plantations involves new investments. Rubber plantation productivity drops significantly when it reaches an age of 30 years, which promotes replanting. Because many households switched their fields from oil palm to rubber plantations in the beginning of the simulation, these plantations need to be replanted at around year 40 (see *Appendix E in* S1 File). With inefficiencies present, mean consumption is more than 50% lower than under efficient production (Fig 5(B, b)). This is partly a direct implication of lower yields due to inefficient production. The heterogeneous inefficiencies also indirectly reduce the number of households who can profit from converting to rubber. When learning is introduced, consumption lies in between the outcome under the efficient and inefficient (without learning) scenarios (Fig 5(C, b)).

The fairly steady improvement of average household welfare is accompanied by relatively constant vegetation carbon dynamics (Fig 5(A, c)). The amount of carbon in the agriculturally used area fluctuates around 35 − 40 tons per hectare within the first 20 simulation years, that is, as long as there is a mixture of oil palm and rubber plantations. During this time, the reduction of vegetation carbon stock due to land-use change is roughly balanced by vegetation growth on those plots where land use does not change. Once all oil palm plantations are replaced by rubber plantations, the vegetation carbon stock increases up to more than 45 tons per hectare, and then slightly decreases again. The decrease in carbon after year 40 is caused by the replanting of old rubber plantations (see *Appendix E in* S1 File). This means that with the applied land-use decision criterion and at the spatial scale and number of households of the model, we observe a tendency towards synchronization, not only of land-use types, but also of plantation ages. This can have both socio-economic and ecological consequences, such as a possible reduction in inequality and amplified cycling in landscape-scale carbon stocks. With the introduction of heterogeneity in inefficiency, carbon stock fluctuations are dampened further, as changes in land use are less dramatic (Fig 5(B, a) and 5(B, c)). When learning is present, trends are again more similar to those under efficient production, albeit with a slower general rate of change (Fig 5(C, c)).

**Fig 6:**
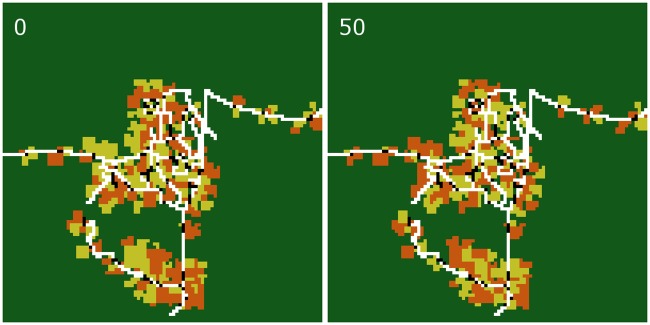
Snapshots of the initial (year 0) and final (year 50) simulated landscape of an exemplary simulation run with historical price trends and no inefficiencies (scenario H0). Roads are marked in white, household home bases in black, oil palm plantations in orange, and rubber plantations in dark yellow. Dark green is the area which is not used for agriculture. An animated representation of this example simulation can be found in Appendix S1 Fig: *Default simulation run animation*.

**Fig 7:**
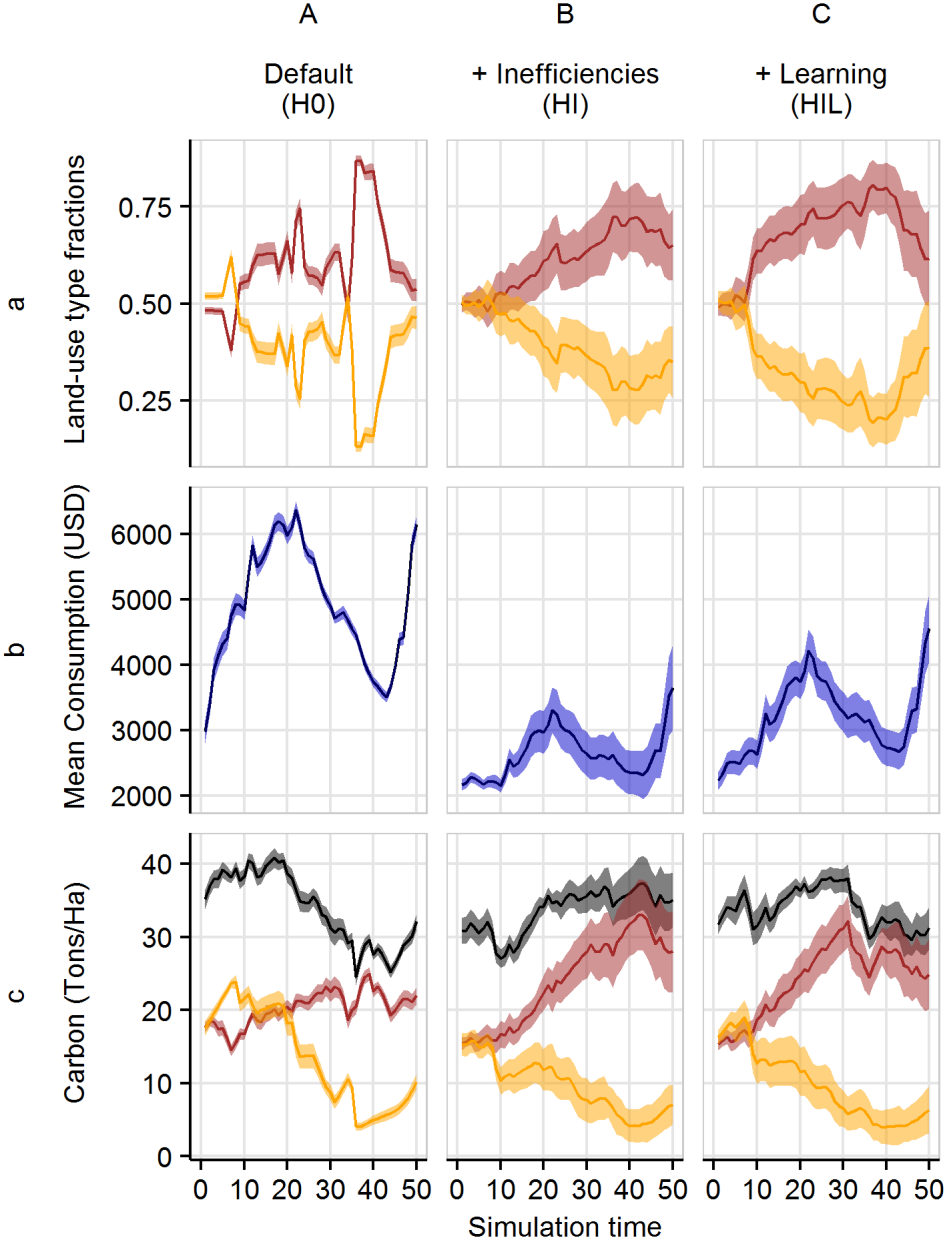
Temporal dynamics under historical price scenarios. Fractions of different land-use types within the agricultural area (a), mean household consumption (b), and vegetation carbon stocks over time (c), with historical price trends; for agents with no inefficiencies and no learning (A), heterogeneous inefficiencies and no learning (B), and heterogeneous inefficiencies and learning (C). In (a) and (c), the light orange color denotes rubber monoculture, while red denotes oil palm. In (c), black refers to the total carbon of land under agriculture. The dark lines represent mean values of the 20 model replications and the shaded polygon surrounding each line represents the respective standard deviations.

When the oil palm and rubber prices fluctuate as with past outputs, the choice of land use no longer settles to a stable state (Fig 6). Instead, when households have no inefficiency, the dominant land-use type varies with the relative changes in the output prices (see Section *Price dynamics, Appendix A in* S1 File). However, not all households are able to switch immediately to the more profitable crop. Households differ in the wealth and size of fields cultivated that originate from initial model conditions. Households with greater wealth and smaller fields (that is, higher investment capacity and lower required investment costs) can be more reactive to price changes and can more easily switch to a new, more profitable land use. Because of the continuous switching, mean household consumption never reaches the levels seen in the scenario with constant output prices (Fig 5); however, similar levels of carbon accumulation under agriculture are reached compared to the constant prices scenarios (Figs 5 and 7). When heterogeneity in inefficiency is included with or without learning, fluctuations in land-use fractions still occur with changing prices, but the overall trend is more stable (Fig 7(B, a) and 7(C, a)). Mean consumption is lower, but peaks occur at similar times as in the scenario with no inefficiencies (Fig 7(B, b) and 7(C, b)). Carbon stored under oil palm and rubber follows trends similar to those of the corresponding land-use fractions (Fig 7(B, c) and 7(C, c)). Interestingly, out of the three historical price trend scenarios, inefficiency without learning resulted in the most stable total carbon stocks at around 35 tons per hectare from year 25 onwards (Fig 7(B, c)).

In order to understand such ecological-economic trade-off dynamics, it is essential to look at the model drivers of carbon stock dynamics. Carbon stocks are driven by land-use change decisions, and land-use change towards the more profitable crop is mainly driven by price dynamics. In the no-inefficiency scenario (Fig 7(A)), households react immediately to changing prices more or less synchronously. In these highly reactive systems, land-use change is quite frequent. The carbon accumulation rates following land-use change (that is, replanting or switching land use of fields) are very low compared to the rates of older plantations (see Section *Carbon storage, Appendix A in* S1 File). Thus, the frequent switching of land-uses results in decreased total carbon stocks. When the efficient production assumption is relaxed and replaced by crop-specific heterogeneous inefficiencies, three types of households appear in the model: those with a lower inefficiency for oil palm than for rubber, those with a lower inefficiency for rubber than for oil palm, and those with similar inefficiencies for both crop types. Households with significant differences in crop inefficiencies focus on the crop type with which they have less inefficiency and only change land use under drastic price changes (see *Appendix E in* S1 File). Additionally, households with high inefficiencies have lower overall yields and are able to generate less capital needed for investment. This makes switching less affordable. The heterogeneity in households and their decision-making reduces synchronization and increases system inertia, which results in a more stable distribution of land-use fractions and thus more stable agricultural carbon stocks (Fig 7(B, c)).

**Fig 8:**
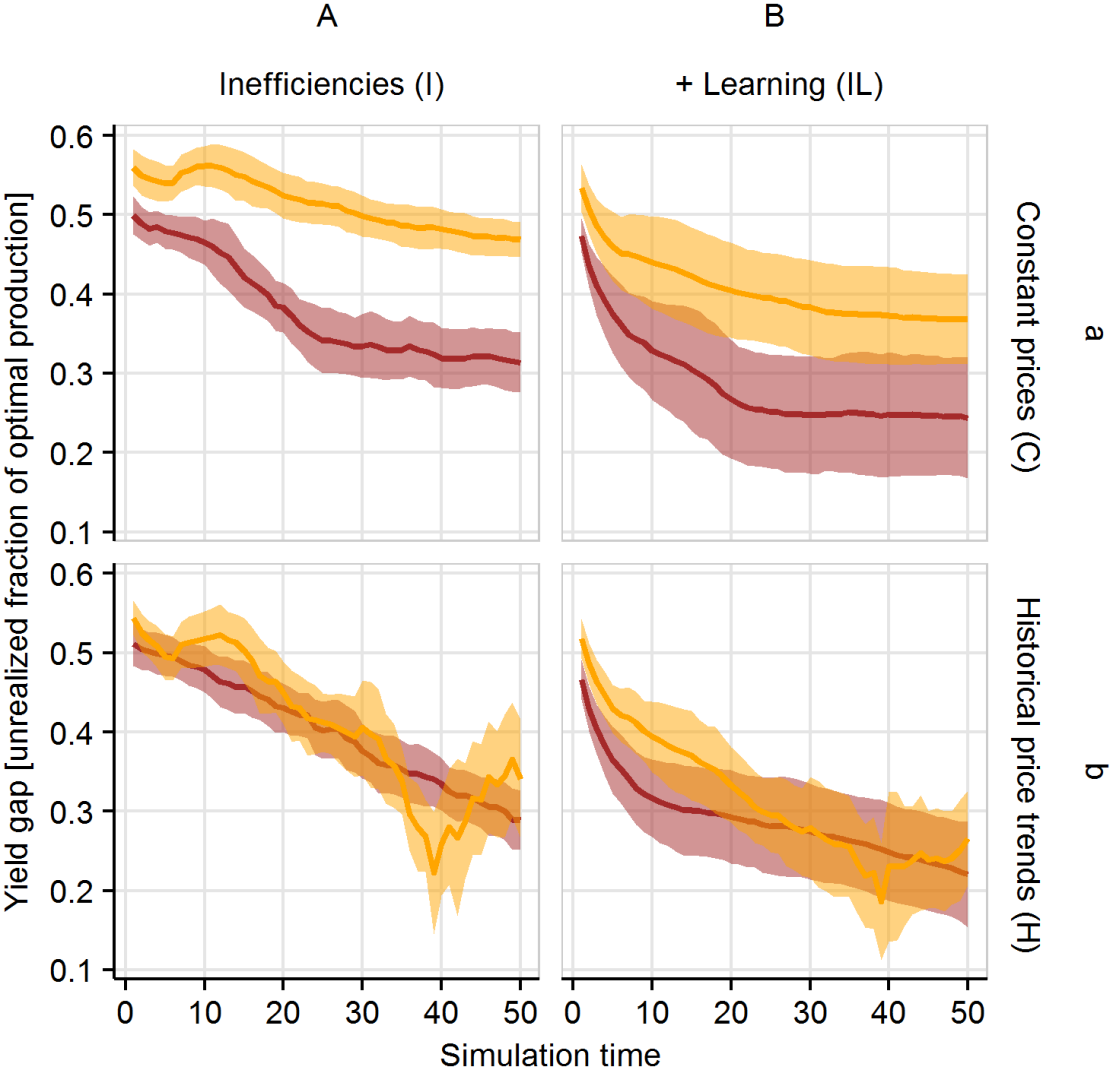
Yield gaps for the scenarios with heterogeneous inefficiencies for constant (CI, CIL) and historical price trends (HI, HIL). Yield gaps are calculated as an unrealized fraction of potential production of all households at each time step for each crop type (yellow: rubber, red: oil palm). Yellow denotes rubber monoculture, while red denotes oil palm. The dark lines represent mean values of the 20 model replications and the shaded polygon surrounding each line represents the respective standard deviations.

To better understand the dynamics at the household level, it is instructive to look at actual yield as a percentage of potential yields. The yield gaps in both crops tend to decline even in scenarios without learning (Fig 8(A, a) and 8(A, b)). The two main reasons for this are: (i) farmers that are very inefficient producers of one crop type tend to switch to the type with which they are relatively more efficient. As very inefficient farmers thus tend to switch first, this reduces the average yield gap for all households active in the production of this crop; (ii) farmers that are inefficient in both crop types tend to take up debts and become frozen early during the simulation.

For example, in the historical price trend scenario (see Section *Price dynamics, Appendix A in* S1 File), the declining initial prices make rubber farming the least profitable to smallholders with high rubber inefficiencies. This lowers the overall yield gap of the remaining rubber farming households down to a minimum at around year 39. Rising rubber prices enables profitability even for households with high inefficiencies, which increases the overall yield gap from year 39 onwards. One might expect to see an inverse trend in oil palm yield gaps, especially in year 39, where we observe an increase in oil palm farming (see Fig 7(B, a) and 7(C, a)). However, oil palm yield gaps show a more or less linear decreasing trend over the entire simulation time. This is because only households that are relatively efficient oil palm producers will switch from rubber to palm oil, while others will take up debts and become frozen if rubber prices are at their minimum (see *Appendix E in* S1 File).

The overall results illustrate the complex interactions between economic and ecological functions, in particular when household heterogeneity is considered. This underlines the need for exploratory tools like EFForTS-ABM. The spatially explicit agent-based approach enabled us to investigate the detailed questions we proposed in the introduction. (1) In most scenarios, and especially in the constant prices scenarios, we do not see clear patterns of trade-offs between household consumption and agricultural carbon stocks. However, smallholder decisions on switching crops and replanting have important impacts on carbon stocks. (2) These decisions are influenced by fluctuating prices. Thus, price dynamics increase heterogeneity in land-use patterns with strong effects on both economic and ecological functions. (3) Productivity heterogeneity among smallholders can have important impacts of ecological-economic trade-offs. Because of the selection processes of more productive farmers (and/or learning), economic benefit can be improved with no harm to carbon storage. However, productivity and heterogeneity also affect the responsiveness of the households to price changes, which, in turn, may not always be beneficial from an ecological perspective.

Regarding our guiding question: “what kind of landscape mosaic can improve the ensemble of ecosystem functioning, biodiversity, and economic benefit based on the synergies and trade-offs that we have to account for?”, we can say that, although carbon storage in oil palm and rubber fields is lower than in primary or secondary forests, the relationships between carbon accumulation/storage and economic benefit are not straightforward when household heterogeneity and switching between crops is taken into account. Rational decisions driven by household characteristics may, for example, lead to ecologically harmful switching that may be avoided by setting up appropriate pooling or compensation mechanisms among households.

## Outlook

The results demonstrate our model’s ability to simulate ecological and economic functions on the basis of a subtle representation of economic decision-making by farmers in a system of two intensified monocultures. At the same time, they demonstrate potentially missing features in the model, such as the lack of leaving land (and plantations) temporarily fallow. Field observations show that farmers are unlikely to immediately switch individual fields from rubber to oil palm production while the rubber is still of a (potentially) profitable age (V. Krishna, personal communication; see also *Appendix D in* S1 File). Furthermore, and along similar lines, observations from the field indicate more inertia in land-use changes than our model suggests. Credit constraints may be more binding than our model assumes or other factors, such as risk (and risk aversion), may prevent farmers from investing in new crops.

More comprehensive treatment of the socio-ecological complexity requires further expansion of the model. Future additions to the model could include large company-owned plantations which might affect knowledge transfer and social networks in our study system. In addition, demographic and land-use dynamics (farm consolidation processes, land markets, migration) need consideration (see [29]).

In terms of the ecological sub-model, further extensions can include soil carbon dynamics (as opposed to just carbon stocks), hydrological functions, and biodiversity of soil microbial communities, plants, insects (including, for example, ants), birds, and possibly mammals [28, 37, 38] (for study designs, see also [8, 30]).

With these additional details, the model will be a flexible tool to examine trade-offs and possible synergies between a multitude of economic benefits and ecosystem services and biodiversity (cf. InVEST modelling tool [39]). For example, similar to Polasky et al. [40], we may then investigate efficiency frontiers, that is, use landscape mosaics where economic benefit is high as starting points to search for alternatives that increase ecosystem services and biodiversity markedly, while having minimal impact on the economic benefits [40, 41].

In summary, Efforts-ABM in its current state serves well as a general tool for exploring the complex interactions and trade-offs between economic smallholder household decisions and ecological functions. Our next steps will include a sensitivity analysis (e.g. [42]), further model refinement, and a validation against observed landscape patterns that will enable us to further increase model usability and reliability.

## Supporting information

S1 File EFForTS-ABM model details. (PDF)

S1 Fig Default simulation run animation. (GIF)

S1 Data Model simulation results. (ZIP)

S2 Data EFForTS-ABM, NetLogo model, and input files. (ZIP)
