# Supplementary material for: Land-use change in oil palm dominated tropical landscapes—An agent-based model to explore ecological and socio-economic trade-offs
Source: PLoS One. 2018 Jan 19;13(1):e0190506. doi: 10.1371/journal.pone.0190506 (PMC5774713; doi:10.1371/journal.pone.0190506)
Supplement: S1 File — (PDF) [file pone.0190506.s001.pdf]

Appendix A. EFForTS-ABM: Model details

1 Implementation details

The land-use change model is implemented in the open source modeling platform NetLogo 5.3.1. The model is still under further development. For questions please contact jsaleck@gwdg.de.

2 Initialization

The most important parts of the initialization are the initial spatial distribution of the different land uses, the location of farming households and the ownership of fields. All these state variables are determined using the landscape generator EFForTS-LGraf (see *S1 Appendix B*) which was developed specifically for this purpose. The outputs of the landscape generator are different raster maps which are read into the land-use change model at the beginning of each simulation run. All raster maps used in the model have a 50m x 50m resolution. The following grid-based maps are used as inputs:

- Forest/nonforest patches
- Land-use-types
- Roads
- Household home-base locations
- Field identity number
- Ownership of cells (connects households to fields)

Exemplary maps which were used for the initialization of the presented model runs are shown in Figure 1 in the main text.

Apart from these initial maps, the following state variables are initialized as follows:

- Initial household wealth is drawn from a log-normal distribution with parameters given in Table S1.3 (see also *S1 Appendix C*). The resulting values for initial wealth are sorted and assigned to households in a way that households owning larger areas have a higher initial wealth.

Table S1.1: List of initial values

Initialization	Unit	Value	Justification
Number of households	[-]	50	
Household area μ (log-normal distribution)	[ha]	1.02	Derived from household data
Household area σ	[ha]	0.91	Derived from household data
Field size μ (log-normal distribution)	[ha]	0.49	Derived from household data
Field size σ	[ha]	0.77	Derived from Household data
Household wealth μ (log-normal distribution)	[USD]	7	Derived from household data
Household wealth σ	[USD]	1	Derived from household data
Household wealth scaling factor	[-]	10	Estimated
Household inefficiency oilpalm α (gamma distribution)	[-]	3.168	Estimated from household data
Household inefficiency oilpalm λ (gamma distribution)	[-]	0.069	Estimated from household data
Household inefficiency rubber α (gamma distribution)	[-]	3.445	Estimated from household data
Household inefficiency rubber λ (gamma distribution)	[-]	0.093	Estimated from household data
Social network median kernel distance	[km]	1	Estimated
Social network maximum kernel distance	[km]	2.5	Estimated
Learning diffusion rate	[-]	0.5	Estimated
Age range of oil palm plantations (uniform distribution)	[year]	[0,30]	Estimated from household data
Age range of rubber plantations (uniform distribution)	[year]	[0,40]	Estimated from household data
Fraction of agricultural area under oil palm and rubber	[-]	0.5:0.5	

- Factors for crop-specific inefficiencies are drawn for each household from gamma distributions (see Section *Initial household inefficiency distribution*). The inefficiency factor reduces potential yields due to lack of expertise or site-specific conditions (see Equation S1.2).

- If learning is turned on, households initialize their social networks based on the spatial proximity to their neighbors (see Section *Household inefficiency & learning*).

- The initial age of agricultural fields is drawn from a uniform distribution with typical age ranges of oil palm and rubber plantations (see Table S1.1).

- The initial carbon contents of fields are set according to their initial ages (see Figure S1.12).

- Initial prices for oil palm fresh fruit bunches (FFB) and rubber as given in Table S1.2 (for details see section *Price dynamics*).

Details on the initialization used for the simulation runs of this paper are given in *S1 Appendix D*, and initial values to variables are summarized in Table S1.1; model parameters are presented in Table S1.2 and Table S1.3. Note that distributions are based on household data [1].

3 Input data

As external input for model initialization, EFForTS-ABM uses maps which are produced by the landscape generator EFForTS-LGraf. Apart from that, the only external variables are the yield prices. Different price functions are implemented (see Section *Price dynamics*), two of which are used in simulations shown in this paper (see Section *Results and Discussion* in the main text).

4 Submodels

The dynamic land-use change model comprises two main sub-models: the economic household submodel that models land-use decisions by rural households and the ecological submodel that simulates ecosystem functions on different spatial scales. In this section we describe the details of these submodels and their parametrization.

4.1 Household model

The economic household model consists of submodels dealing with household production and capital accumulation (see Section *Production function, cash flows and capital accumulation*) as well as the corresponding land-use change decisions (see Sections *Decision on land-use change and production* and *Reduction of factor inputs*). In short, the economic household model includes the following processes (Fig. 3 in the main text). At the beginning of each time step, if "learning" is turned on, households simultaneously improve their productivity by decreasing their inefficiencies (see Section *Household inefficiency & learning*). Then household wealth is reduced by its planned consumption (box Consumption I in Fig. 3). Each household then decides on land management (box Land management in Fig. 3) including the decision on factor inputs and land-use change. This decision is based on the expected cash flows from different land use options over a certain time horizon (e.g. 10 years). We assume that households are credit constrained. This means that households might not be able to realize the most profitable land-use option, as they might, for example, not be able to mobilize the capital necessary for initial investment. Following the land management decision, annual yields (Yield in Fig. 3) of all household cells are calculated. Yields are affected by the age of plantations, factor inputs and household inefficiency, reflecting inefficient knowledge and site-specific conditions. Given current output prices (Output prices) the realized annual revenue (Revenue) is derived. Given

current factor prices (Factor prices), costs (Costs) for agricultural production are calculated and subtracted from the revenue, resulting in the annual cash flow (Cash flow) of the household. In the case of positive annual cash flow, a part of the cash flow is consumed (Consumption II). The household's wealth (Wealth/Savings) is updated by adding the remaining cash flow and external income. The updated household wealth influences which land-use options are feasible for the household in the next time step.

4.1.1 Production function, cash flows and capital accumulation

For each household cell j we apply a Leontief production function [2]. This implies that factors cannot be substituted and production is determined by the input factor which is applied in the smallest relative amount. Thus, production is calculated as

$$\hat{y}_{j,l,n}(L, K, TI, \overline{LA}) = min\left\{ y_n^* \frac{L}{L_n^*}, y_n^* \frac{K}{K_n^*}, y_n^* \frac{TI}{TI_n^*}, y_n^* \frac{\overline{LA}}{\overline{\overline{LA}}} \right\} \qquad \text{(S1.1)}$$

with
$\hat{y}_{j,l,n}$: production [ton] from crop l of age n on cell j under the factor inputs labor L, capital K, technical inputs TI and land $\overline{LA}$
y_n^*: production [ton] of a plantation of age n on one cell with optimal factor inputs (see section *Production functions for oil palm and rubber* for the derivation of the optimal production)
L_n^*: the optimal factor input of labor [hour] for a plantation of age n
K_n^*: the optimal capital stock [US$] for a plantation of age n
TI_n^*: the optimal factor input of technical input [US$] for a plantation of age n
$\overline{LA}$: Land [ha], which is fixed to the size of one cell.

The Leontief production function defines the potential production given a certain age of a plantation and certain levels of inputs (for oil palm plantations, yield is calculated in tons of fresh fruit bunches per hectare and year; rubber yield is calculated in tons of rubber per hectare and year). However, due to varying experience of farmers in the cultivation of different land uses, incomplete knowledge, e.g. about ideal timing of fertilization or harvesting, as well as variation in site-specific conditions, this potential production might not be realized by a household. We incorporate the gap between potential and realized yield by introducing an inefficiency factor $\delta_{i,l}$ for each

household i and land-use type l. The realized production from cell j which is owned by household i is therefore

$$y_{j,l,n}(L,K,TI,\overline{LA}) := \delta_{i,l} \cdot \hat{y}_{j,l,n}(L,K,TI,\overline{LA}) \ . \tag{S1.2}$$

Based on the assumption that input factors are the same for all cells belonging to one field, the production for a field consisting of m cells of crop l of age n is given by

$$yfield_{l,n} = y_{j,l,n}(mL,mK,mTI,m\overline{LA}) = m \cdot y_{j,l,n}(L,K,TI,\overline{LA}) \ . \tag{S1.3}$$

The revenue [US$] from cell j in year t is

$$Rcell_{j,t} = y_{j,l,n_t}(L,K,TI,\overline{LA}) \cdot p_{l,t} \tag{S1.4}$$

with
n_t: the age [year] of the plantation in cell j at time t
$p_{l,t}$: price [US$/ton] of the product of land use l at year t.

The total revenue [US$] from agricultural land use of household i in year t is thus given by

$$R_{i,t} = \sum_{household\ cells\ j} Rcell_{j,t} \ . \tag{S1.5}$$

The net cash flow [US$] from cell j in year t is

$$\Pi cell_{j,t} = Rcell_{j,t} - rcost_{j,l,n_t}(L,K,TI,\overline{LA}) - icost_{j,l,n_t} \tag{S1.6}$$

with
$rcosts_{j,l,n_t}$: recurrent costs [US$] on cell j under crop l in year t, depending on factor inputs of labor L, capital K, technical inputs TI and land $\overline{LA}$
$icost_{j,l,n_t}$: investment costs on cell j for agricultural production of crop l in period t.

The net cash flow [US$] from agricultural land use for household i in year t is thus given by

$$\Pi_{i,t} = \sum_{household\ cells\ j} \Pi cell_{j,t}\ . \tag{S1.7}$$

The recurrent costs for cell j are calculated as

$$rcost_{j,l,n_t}(L,K,TI,\overline{LA}) = \begin{cases} r_{K,t}K + r_{L,t}\overline{LA}\ , & \text{if } n_t < n_m \\ w_{l,t}L + r_{K,t}K + p_{TI,l,t}TI + r_{L,t}\overline{LA} & \text{if } n_t \geq n_m \end{cases} \tag{S1.8}$$

with
n_t: the age of the plantation on cell j at time t
n_m: the maturation age of the plantation, i.e. the first year with non-zero yields
$r_{K,t}$: rental rate of capital in year t
K: the current capital stock [US$] on cell j
$r_{L,t}$: rental rate of land [US$/ha] in year t (independent of what crop is on the cell)
$w_{l,t}$: wage for one hour of work [US$/h] in crop l in year t
L: input of labor [hour] on cell j in year t
$p_{TI,l,t}$: price for one unit of technical input [US$/kg] in crop l at time t[1]
TI: technical input [kg] on cell j in year t.

We assume that investment costs occur only within the immature phase of a plantation life cycle, i.e. as long as yields are zero. The total investment costs $icost\_total_{j,l}$ for a plantation of crop l in one cell j are therefore

$$icost\_total_{j,l} = \sum_{k=0}^{n_m-1} icost_{j,l,k} \quad . \tag{S1.9}$$

These investment costs include non-recurrent costs, e.g. for buying seedlings, as well as all costs for labor and technical input in the immature phase. For establishing oil palms, for example, labor is needed for lining, the transportation of seedlings, and digging holes. Land is already owned by the household, i.e. part of its initial endowments, and we only consider the opportunity costs of holding this asset. During the immature period, the capital stock is built up and we assume that no further investment costs occur once positive yields are produced. From this point onwards all labor

[1] Both wages and prices for technical input as well as rental rates for capital and land can vary with time. However, in our current model version, we keep them fixed and therefore omit the index t in the remainder of the model description.

and input costs are classified as recurrent costs. We acknowledge that some of these recurrent costs could similarly be conceptualized as maintenance, i.e. reinvestment costs, but our simplification facilitates modeling of the crop choice decision later on.

Each household cell j has a capital stock $K_{j,t}$, representing the resale value of the capital stock embodied in rubber trees or oil palms on the cell at time t (see Eq. (S1.1) and (S1.8)). The capital stock is calculated as the cumulative investment costs in this cell minus depreciation

$$K_{j,t} = (1 - d_l(n_t)) \cdot K_{j,t-1} + icost_{j,l,n_t} \tag{S1.10}$$

with depreciation rate $d_l(n_t)$. The depreciation rate, which captures the natural productivity of the plantation, depends on the plantation age n_t: for young plantations, d is negative, for older ones positive. This is because productivity generally increases in young plantations and decreases in old plantations; the productivity inflection point is crop-specific. The total capital stock of household i in year t is accordingly

$$Ktot_{i,t} = \sum_{household\ cells\ j} K_{j,t} \ . \tag{S1.11}$$

4.1.2 Decision on land-use change and production

The decision on land management and production, i.e. land-use change and the corresponding factor inputs, is determined by the profitability of land use options, as well as wealth (and consumption) of the household. Let $W_{i,t-1}$ be the wealth of household i at the end of year $t-1$, i.e. the wealth available at the beginning of year t. For simplicity we assume, that, apart from the profit-based component of household consumption, all expenditures occurring in year t need to be disbursed by the household, i.e. paid before the income from agricultural production and external sources in the year t is available. Household consumption is calculated in a two-step process, partly before and partly after net cash flow realization of that time step. The planned household consumption of household i, $C\_plan_{i,t}$ consists of a fixed base consumption $\bar{C}_i$ representing the subsistence level, and a variable additional consumption depending on the actual wealth $W_{i,t-1}$.

Thus the planned consumption of household i in year t is

$$C\_plan_{i,t} = \bar{C}_i + C_W \cdot W_{i,t-1} \tag{S1.12}$$

with C_W the fraction of wealth that is additionally consumed.

The actually realized consumption $C_{i,t}$ can increase by a profit-based component, if a positive net cash flow in this year permits additional consumption (see Table S1.3 for parameter values of consumption). Thus, after the calculation of the net cash flow $\Pi_{i,t}$, household consumption is updated according to

$$C_{i,t} = \begin{cases} C\_plan_{i,t} + C_\pi \cdot \Pi_{i,t} & \text{, if } \Pi_{i,t} > 0 \\ C\_plan_{i,t} & \text{, if } \Pi_{i,t} \leq 0 \end{cases} \quad . \tag{S1.13}$$

The wealth after planned consumption is available to cover investment and recurrent costs of agricultural production. We define a minimum wealth level W_{min} that is always available to a household, assuming that the household can, if necessary, cover costs for consumption from a safety net (family, friends, etc. as a short term credit). Therefore the available resources for factor inputs and land-use change in year t are

$$W_{i,temp} := \begin{cases} W_{i,t-1} - C\_plan_{i,t} & \text{, if } W_{i,t-1} - C\_plan_{i,t} \geq W_{min} \\ W_{min} & \text{, else} \end{cases} \tag{S1.14}$$

If the actual household wealth does not cover the planned consumption, the household temporarily takes up debts $D_{i,t,temp}$ of the amount

$$D_{i,t,temp} := C\_plan_{i,t} + W_{min} - W_{i,t-1} \quad . \tag{S1.15}$$

In each period t each household decides on management of its household fields after reducing the wealth by the annual household's planned consumption (see Eq. S1.14). This decision includes the decisions on factor inputs and land-use changes, which are taken simultaneously. It depends on the available capital for agricultural production $W_{i,temp}$.

Since we consider two possible land-use types (oil palm and rubber plantation), there are three possible options for each household field: to continue the actual land-use, to replant the actual land-use type or to change to the alternative land use. If a household has u fields, the number of possible options is thus 3^u. As the calculation of expected cash flows from different land-use options is the most time-consuming part of the model, we implemented two versions of this process: the "all-fields"-option, which allows the full number of options, i.e. in principal a change of land use in all fields of a

household within one year, and a “one-field-per-year”-option in which each household can change only one field per year. The latter reduces the number from 3^u to $3u$. The option can be chosen on the GUI. For this paper, we apply the one-field-per-year option.

From the set of all options, only those are potentially possible, for which total investment costs (i.e. investment costs from all household fields within the next three years) as well as unavoidable recurrent costs in the current year can be covered by the actual wealth $W_{i,temp}$ (see Eq. S1.17) while not falling under the minimum wealth level.

Let o be an arbitrary option, p_k the fields of the household ($k = 1, ...g$) and let l_k be the intended land uses on these fields under option o. Let furthermore m_k be the field sizes (i.e. number of cells in the field). The discounted total investment costs under the option o within the next three years are

$$^{o}Itot_i := \sum_{household\ fields\ p_k} \left(m_{p_k} \cdot \sum_{n=0}^{2} icost_{j_{p_k}, l_{p_k}, n_t+n} \cdot (1+r)^n \right), \quad \text{(S1.16)}$$

with j_{p_k} a representative cell of field p_k, l_{p_k} the intended land use on field p_k, n_t the age of field p_k at time t and discount rate r.

Therefore, if

$$W_{i,temp} \geq^{o} Itot_i + \underbrace{\sum_{household\ cells\ j} (r_K K^{*}_{n_t,j} + r_L \overline{LA})}_{\text{unavoidable recurrent costs}} + W_{min}\ , \quad \text{(S1.17)}$$

option o can potentially be afforded by the household. This is a simplifying assumption as it neglects that a household could potentially cover the investment costs of the second and third year by the income in these years from other fields. If no option is affordable, the household chooses the “no change” option, i.e. all land uses remain the same and no replanting takes place.

The following steps are executed for each affordable option with the goal to choose the most profitable one.

In the current year t, investment costs due to the implementation of option o are

$$\sum_{household\ fields\ p_k} m_{p_k} \cdot icost_{j_{p_k},l_{p_k},n_t} \ . \tag{S1.18}$$

Therefore, if option o is implemented, the remaining capital available for factor inputs in year t is

$$^{o}W_{i,rest} := W_{i,temp} - \sum_{household\ fields\ p_k} m_{p_k} \cdot icost_{j_{p_k},l_{p_k},n_t} \geq 0 \ . \tag{S1.19}$$

If the remaining capital $^{o}W_{i,rest}$ is sufficient for optimal factor input on all fields, i.e. if

$$^{o}W_{i,rest} \geq \sum_{household\ fields\ p_k} m_{p_k} \cdot rcost_{j_{p_k},l_{p_k},n_t}(L^*_{n_t}, K^*_{n_t}, TI^*_{n_t}, \overline{LA}) \ , \tag{S1.20}$$

and no additional external constraints are existent, the household will apply optimal factor inputs to maximize production and profit from agricultural land use. If the remaining capital is not sufficient for optimal factor inputs, i.e.

$$^{o}W_{i,rest} < \sum_{household\ fields\ p_k} m_{p_k} \cdot rcost_{j_{p_k},l_{p_k},n_t}(L^*_{n_t}, K^*_{n_t}, TI^*_{n_t}, \overline{LA}) \ , \tag{S1.21}$$

factor inputs are reduced (see section *Reduction of factor inputs*). Net cash flow $^{o}\Pi_{i,t}$ under option o and actual factor inputs is calculated. The fictive household wealth under application of option o is updated to

$$^{o}W_{i,t} = W_{i,temp} +^{o} \Pi_{i,t} + \tilde{Y} \tag{S1.22}$$

with external household income $\tilde{Y}$.

To decide which of the affordable options should be chosen by the household, we calculate the expected cash flow from agricultural use within a certain time horizon h for each potential option o. For this we also need to calculate the expected factor inputs during that time. As optimal factor inputs vary with plantation age and actual factor inputs depend on wealth, we need to simulate the wealth development of the household over the given time horizon. For this we assume, that within this period of h years no more land-use

changes occur.

Prices for input, output and labor are assumed to stay constant within the considered time horizon and at the level of prices in period t. Also the external income is assumed to stay the same as in year t. Household consumption for each year is calculated based on the expected wealth in the respective year. Let ${}^{o}\Pi_{i,t}$,...,${}^{o}\Pi_{i,t+h}$ be the expected net cash flows from agricultural use under option o within the time horizon h. For each option o the discounted accumulated expected cash flow is calculated as

$$ {}^{o}\Pi\_expected_i := \sum_{j=0}^{h} \frac{{}^{o}\Pi_{i,t+j}}{(1+r)^j} \ , \tag{S1.23} $$

with discount rate r. The option with the maximal expected cash flow is then implemented.

4.1.3 Reduction of factor inputs

In the case of Equation S1.21 the household cannot afford optimal factor inputs if option o is implemented. Therefore, factor inputs need to be reduced. However, Equation S1.17 assures that the unavoidable rental costs for capital and land can be covered as

$$ {}^{o}W_{i,rest} \geq \sum_{household\ cells\ j} (r_K K^*_{n_{t,j}} + r_L \overline{LA}) + W_{min} \ . \tag{S1.24} $$

We assume that costs for capital and land are fixed and only the input factors labor L and technical input TI can be reduced. The amount of available resources for factor input is

$$ {}^{o}W_{i,FI} :={}^{o} W_{i,rest} - \sum_{household\ cells\ j} (r_K K^*_{n_{t,j}} + r_L \overline{LA}) \ . \tag{S1.25} $$

To determine on which fields factor inputs are reduced, the marginal loss for a representative cell j_m of each household field m is calculated. Factor inputs are reduced on the fields with lowest marginal losses, until all remaining capital is used.

The production of one unit of output less involves less labor and technical input and thus reduces the costs by an amount of *cost_red*. Since we apply a Leontief production function, each unit of production in a plantation of

age n involves factor inputs of L^*_n/y^*_n of labor and TI^*_n/y^*_n of technical input, where L^*_n and TI^*_n are the optimal factor inputs to produce the maximum output y^*_n in a plantation of age n. Therefore, the optimal factor input for the production of $y^*_n - 1$ output units on one cell is

$$\left(L^*_n - \frac{L^*_n}{y^*_n}, K^*_n, TI^*_n - \frac{TI^*_n}{y^*_n}, \overline{LA})\right) \ . \tag{S1.26}$$

The cost reduction involved in producing one unit of output less on one cell is thus

$$\begin{aligned} cost\_red &= rcost(L^*_n, K^*_n, TI^*_n, \overline{LA}) - rcost\left(L^*_n - \frac{L^*_n}{y^*_n}, K^*_n, TI^*_n - \frac{TI^*_n}{y^*_n}, \overline{LA})\right) \\ &= w \cdot \frac{L^*_n}{y^*_n} + p_{TI} \cdot \frac{TI^*_n}{y^*_n} \end{aligned} \tag{S1.27}$$

with
w: wage for one hour of work [US$/h]
p_{TI}: price for one unit of technical input [US$/kg].

The marginal loss ($mloss$) in net cash flow from cell j under land use l is thus

$$\begin{aligned} mloss &= \Pi cell_{j,t}(L^*_n, K^*_n, TI^*_n, \overline{LA}) - \Pi cell_{j,t}\left(L^*_n - \frac{L^*_n}{y^*_n}, K^*, TI^*_n - \frac{TI^*_n}{y^*_n}, \overline{LA}\right) \\ &= p_{l,t} - cost\_red \end{aligned} \tag{S1.28}$$

with
$p_{l,t}$ the revenue for one unit of production (= price [US$/ton] of product of land use l in year t.).

Those fields with high marginal losses should receive optimal factor input, if possible. Therefore factor inputs are determined starting with the field with the highest marginal loss. Let p be the field with the highest marginal loss, m be the number of cells in p and n_p the age of the plantation in field p.

If the remaining resources for factor inputs ${}^oW_{i,FI}$ cover the costs for optimal input of labor and technical input on field p, i.e.

$${}^oW_{i,FI} \geq m \cdot (wL^*_{n_p} + p_{TI}TI^*_{n_p}) \ , \tag{S1.29}$$

this field will receive optimal factor input and ${}^oW_{i,FI}$ is reduced by these costs:

$$ {}^oW_{i,FI} := {}^o W_{i,FI} - m \cdot (wL^*_{n_p} + p_{TI}TI^*_{n_p}) \ . \tag{S1.30} $$

This process is continued for the other household fields with decreasing marginal loss until the field is reached at which the remaining resources ${}^oW_{i,FI}$ are not sufficient anymore to cover optimal factor inputs.

Let q be this field of size m_q and age n_q, where

$$ {}^oW_{i,FI} < m_q \cdot (wL^*_{n_q} + p_{TI}TI^*_{n_q}) \ . \tag{S1.31} $$

As each unit of production involves labor and technical input costs of

$$ w \cdot \frac{L^*_{n_q}}{y^*_{n_q}} + p_{TI} \cdot \frac{TI^*_{n_q}}{y^*_{n_q}} \ , \tag{S1.32} $$

the household can afford a production of

$$ f := {}^o W_{i,FI} \Big/ \left(w \cdot \frac{L^*_{n_q}}{y^*_{n_q}} + p_{TI} \cdot \frac{TI^*_{n_q}}{y^*_{n_q}} \right) \tag{S1.33} $$

units. The factor inputs for labor and technical input on this field are thus

$$ f \cdot \frac{L^*_{n_q}}{y^*_{n_q}} \quad \text{and} \quad f \cdot \frac{TI^*_{n_q}}{y^*_{n_q}} \ . \tag{S1.34} $$

The remaining fields do not receive inputs of labor or technical inputs in this year.

At the end of this step, factor inputs for each household cell are known. Thus the profit from land use under option o with these factor inputs can be calculated for each household cell and household wealth can be updated according to Equation S1.22.

4.1.4 Implementation of the land management decision

Now it is clear which of the affordable options is implemented and also the factor inputs are known. Let o be the chosen option, then the unavoidable costs in this year are potential investment costs as well as the recurrent costs for capital and land

$$^{o}costs_u := \sum_{household\ cellj} ({}^{o}icost_j + r_t K_j + r_{L,t}\overline{LA}) \quad . \tag{S1.35}$$

Similar to Equation S1.14, these unavoidable costs are subtracted from the current wealth, respecting the minimum wealth level

$$W_{i,temp2} := \begin{cases} W_{i,temp} -^{o} costs_u & \text{, if } W_{i,temp} -^{o} costs_u \geq W_{min} \\ W_{min} & \text{, else.} \end{cases} \tag{S1.36}$$

If the household needs to take up debts to assure the minimum wealth level, these debts amount to

$$D_{i,t,temp2} :=^{o} icost_j + W_{min} - W_{i,temp2} \quad . \tag{S1.37}$$

Finally, the factor inputs of labor and technical inputs under option o reduce the wealth

$$W_{i,temp3} := W_{i,temp2} - ({}^{o}cost_{L,t} +^{o} cost_{TI,t}) \quad , \tag{S1.38}$$

with
${}^{o}cost_{L,t}$ costs for labor in year t under option o,
${}^{o}cost_{TI,t}$ costs for technical inputs in year t under option o.

Any debts a household gets into in the current year, e.g. due to consumption or due to unavoidable costs (see Eq. S1.15 and S1.37), are added to the potentially remaining debts from the previous year, and if possible, payed off at the end of the period, when cash flows are realized. Household debts in period t before pay off are therefore

$$D_{i,temp3} := D_{i,t-1} + D_{i,t,temp} + D_{i,t,temp2} \tag{S1.39}$$

with
$D_{i,t-1}$ debts after pay off in period $t-1$.

Now the cash flow from the realized option o as well as the external income are added to the household wealth and the cash flow dependent part of consumption is accounted for

$$W_{i,temp4} := \begin{cases} W_{i,temp3} + (1-\beta)^{o}\Pi_{i,t} + \tilde{Y}_i & \text{, if } {}^{o}\Pi_{i,t} > 0 \\ W_{i,temp3} +^{o} \Pi_{i,t} + \tilde{Y}_i & \text{, if } {}^{o}\Pi_{i,t} \leq 0 \end{cases} \tag{S1.40}$$

with
${}^{o}\Pi_{i,t}$ the cash flow in this year,
$\tilde{Y}_i$: external income in year t,
β the cash flow dependent fraction of consumption (see Eq. S1.13).

Finally, the household pays off debts but respects the minimum wealth level. Therefore the household wealth which is available for the next year is

$$W_{i,t} := \begin{cases} W_{i,temp4} - D_{i,t,temp3} & \text{, if } W_{i,temp4} - D_{i,t,temp3} > W_{min} \\ W_{min} & \text{, if } W_{i,temp4} - D_{i,t,temp3} \leq W_{min} \end{cases} . \tag{S1.41}$$

The household debts are updated accordingly to

$$D_{i,t} := \begin{cases} 0 & \text{if, } W_{i,temp4} - D_{i,t,temp3} > W_{min} \\ D_{i,temp3} - (W_{i,temp4} - W_{min}) & \text{if, } W_{i,temp4} - D_{i,t,temp3} \leq W_{min} \end{cases} \tag{S1.42}$$

Households which do not manage to pay back debts within a certain period, i.e. $D_{i,t} > 0$ for D_{max} consecutive years (see Table S1.2), are assumed to be incapable of acting and are frozen in the model.

4.1.5 Household inefficiency & learning

Depending on the parameterization of the model, households can have crop-specific heterogeneous inefficiency values for oilpalm and rubber (see section *Initial household inefficiency distribution*). The higher the inefficiency value of a specific land use type is, the lower is the realized yield, compared to the optimal production function (see Eq. S1.2).

If "learning" is turned off, households inefficiency values do not change over time, assuming no diffusion of knowledge or spillover effects. However, if "learning" is turned on, households can improve their productivity by decreasing their inefficiency values over time. This follows the assumption, that social networks and interactions between households lead to improvements in farming efficiency. The creation of the social network is executed once during the initialization of the model. In the current model version, this social network is a static network and does not change over time. The model offers four options to create the social networks of households. (1) The network is created by connecting each household with its n-nearest-neighbors based on the road distances. This approach is deterministic and assuming all households are connected via the same road network, each

household has the same number of connected households. However, the diffusion of information inside the network can still differ depending on the initial distribution of inefficiencies. (2) The network is created by connecting each household, with every household that can be reached within a specified road distance. This approach is deterministic and households are likely to be heterogeneous in numbers of connected households, depending on the spatial proximity to other households. (3) The network is created by using a negative exponential function, that serves to calculate household distance connectivity probabilities. The probability function is scaled to an estimated median household connectivity distance. Then, for each pair of two households that is connected via roads, the geographic distance via the road network is calculated. The two households are then connected, based on the distance specific connection probability, derived from the negative exponential household distance probability function. This approach is non-deterministic and households are likely to be heterogeneous in numbers of connected households. (4) The network is created by using the negative exponential function (3) in combination with a maximum distance bound (2).

Learning takes place at the beginning of each simulation step (see Fig. 2). Households are only allowed to improve inefficiencies for crops they currently cultivate. Furthermore, they can only learn from connected households that cultivate this crop as well. For each specific crop, each household calculates the mean of crop inefficiencies of connected households that fit the given requirements. The mean inefficiency of connected households for this crop type, represents the mean inefficiency of the households social network. If the household inefficiency for a specific crop type is higher than the mean inefficiency of its social network, the household can improve its inefficiency for that crop type. The level of improvement is calculated by multiplying the difference of household inefficiency and its social network inefficiency with the parameter "learning-diffusion-rate", that defines the speed of household adjustment, i.e. diffusion rate of the social network. If "learning-diffusion-rate" is set to 1, all households immediately adjust to the mean inefficiency level of their social networks.

4.2 Parametrization of the household submodel

For the implementation of the Leontief production function, we consider the following economic functions: optimal production, optimal labor use, optimal amount of technical inputs, optimal capital stock, and the use of land. Apart from land, all economic functions depend on the age of the respective

plantation. To derive these functions and their parameters we used data from a household survey in the province of Jambi, Sumatra [3, 4]. Jambi is the focus of the Collaborative Research Center EFForTS (Ecological and Socioeconomic Functions of Tropical Lowland Rainforest Transformation Systems (Sumatra, Indonesia)) which has started in 2012. Interdisciplinary research on social and economic dynamics has provided a household survey of 701 households, which include information such as households' land holdings, agricultural and non-agricultural activity, endowments and household composition (for more details see [5, 1, 6, 7]). The survey represents a random sample out of 40 villages which in return are randomly chosen out of 5 regencies within the province of Jambi. The respective sample sizes per village are chosen proportionally to village size. Out of the household sample, we use information on the production of 246 oil palm farmers cultivating 385 oil palm fields and 579 rubber farmers cultivating 962 rubber fields. Drawing on the reported ages of plantations, the oil palm fields of oil palm farmers are between 0 and 23 years old and the rubber fields have an age between 0 and 45 years. This enables a data-based parametrization of the economic functions for these time spans. Since we do not assume a maximum plantation age in our model, we also need to extrapolate economic functions for plantation ages beyond the data. To derive the production function, we estimate optimal yield, labor and technical inputs. For the estimation of optimal yields we selected the 30% highest yielding fields per plantation age ($N = 105$ for oil palm and $N = 244$ for rubber) (see Fig. S1.1 (a) and (b)). Assuming that these fields are optimally managed, they were also used to derive model functions and parameters for optimal labor and technical input.

4.2.1 Production functions for oil palm and rubber

Optimal production
Yields of the 30% highest yielding oil palm and rubber fields is presented in Figure S1.1 (a,b). As an estimation of the optimal, i.e. maximal potential fresh fruit bunch production over palm age, we derived a function which reproduces the bunch production of the process-based PALMSIM model, which was validated against 13 sites in Indonesia and Malaysia [8, see Fig. S1.1 c]. After the immature phase of three years, in which yield is zero, this function has a roughly exponential increasing phase, which is followed by a plateau and a decreasing yield phase. The applied function is

$$production_{oil\ palm}(x) = \begin{cases} 0 \text{ , if } x \leq 2 \\ p_{o1} \cdot exp(p_{o2} \cdot x) \text{ , if } 2 < x \leq 7 \\ p_{o3} \text{ , if } 7 < x \leq 11 \\ max\{0, p_{o4} \cdot x + p_{o5}\} \text{ , if } x > 11 \end{cases} \quad \text{(S1.43)}$$

with parameters shown in Table S1.2. As we do not assume a maximum plantation age in our model, this function is also used to extrapolate production for plantation ages beyond the data (see Fig. S1.1 e).

For rubber, we estimated the potential yield from our data and used a parabola which reflects the limited life span of tapped rubber trees. As we are interested in the maximal possible yields, we require rather an envelope function above the data than a fit. Therefore, we shift the fitted function upwards so that 95% of the data from high yielding fields are under the curve (Fig. S1.1 (d)). We fix the production of rubber in the first five years to zero. The resulting optimal production function for rubber is shown in Figure S1.1 (f). The applied optimal production function for rubber is therefore

$$production_{rubber}(x) = \begin{cases} 0 \text{ , if } x \leq 4 \\ max\{0, p_{r1} \cdot x^2 + p_{r2} \cdot x + p_{r3}\} \text{ , if } x > 4 \end{cases} \quad \text{(S1.44)}$$

with parameters shown in Table S1.2.

Optimal labor input

To estimate optimal labor use we draw on the labor data from the same 30% highest yielding fields per plantation age, but exclude data from the first three years for oil palm, and respectively the first five years for rubber, as we consider input of labor during this period as part of the investment. The data on labor comprise operations such as land clearing, pits taking, seedling transportation, planting and replanting, manure and fertilizer application, chemical and manual weeding, harvesting, pruning and marketing. Working hours per hectare are accumulated for each best performing field. The data are very scattered for both land uses (see Fig. S1.2 (a) and (b)). For oil palm an increase in labor after the plantation establishment phase followed by a slight decrease in labor input is apparent. We tested different relationships: a hump-shaped function

$$lab(x) = l_1 + \frac{x}{l_2} exp^{-\frac{x}{l_2}} \quad , \quad \text{(S1.45)}$$

Table S1.2: Parameters of the economic household model related to the Leontief production function and costs.

Category	Land-use type	Parameter	Unit	Meaning	Value	Reference/Justification
Production	Oil palm	p_{o1}	[-]	Scaling (exponential growth phase)	0.3	
		p_{o2}	[-]	Exponent (exponential growth phase)	0.7	
		p_{o3}	[T]	Plateau value (plateau phase)	40	[8]
		p_{o4}	[-]	Slope (decreasing phase)	-0.6	
		p_{o5}	[T]	Intercept (decreasing phase)	46	
		p_o	[USD/ton]	Price fresh fruit bunches	90	Household data
	Rubber	p_{r1}	[-]	Quadratic parameter of parabola	-0.007	Household data
		p_{r2}	[T]	Linear parameter of parabola	0.3	Derived from household data
		p_{r3}	[-]	Constant parameter of parabola	2.5	
		p_r	[USD/ton]	Price rubber	1100	Household data
Labour	Oil palm	l_{o1}	[y]	Breakpoint 1	5	
		l_{o2}	[year]	Breakpoint 2	7	
		l_{o3}	[year]	Breakpoint 3	25	
		l_{o4}	[h/year]	Slope segment 1	100	
		l_{o5}	[h/year]	Slope segment 2	-80	Derived from household data
		l_{o6}	[h/year]	Slope segment 3	-0.8	
		l_{o7}	[h]	Intercept segment 1	-230	
		l_{o8}	[h]	Intercept segment 2	690	
		l_{o9}	[h]	Intercept segment 3	120	
		l_{o10}	[h]	Plateau value (old plantations)	1400	Calibrated
	Rubber	l_{r1}	[h/(ha year)]	Labour input (plantation age > 4)	700	Derived from household data
	All land-use types l	w_l	[USD/hour]	Wage	1.6	Household data
Technical input	Oil palm	t_{o1}	[kg/(ha year)]	Constant input mature phase	740	Household data
	Rubber	t_{r1}	[kg/(ha year)]	Constant input mature phase	150	Household data
	All land-use types l	p_{TI}	[USD/kg]	Price technical input	0.5	Household data
Capital	Oil palm	$icost\_total_o$	[USD/ha]	Investment costs immature phase m	[600 200 150]	Household data
		d_{o2}	[-]	Depreciation rate young plantations	-0.1	
		d_{o3}	[-]	Depreciation rate old plantations	0.1	Estimated
		c_{o4}	[year]	Age in which depreciation rate switches	10	
	Rubber	$icost\_total_r$	[USD/ha]	Investment costs immature phase m	[200 70 70 70 70]	Household data
		d_{r2}	[-]	Depreciation rate young plantations	-0.05	
		d_{r3}	[-]	Depreciation rate old plantations	0.05	Estimated
		c_{r4}	[year]	Age in which depreciation rate switches	15	
	All land-use types	r_K	[-]	Rental rate of capital	0.1	Estimated
Land	All land-use types l	r_L	[-]	Rental rate of land	0.1	Estimated
		p_L	[USD/ha]	Land price	750	Household data
Debts	All land-use types l	D_{max}	[-]	Number of consecutive years households in debt are frozen	5	Estimated

Table S1.3: Parameters of the economic household model related to household wealth and consumption.

Parameter	Unit	Meaning	Value
W_{min}	[$]	Minimum wealth level	30
D_{max}	[year]	Maximum number of consecutive debt years	5
$\tilde{Y}$	[$]	External annual household income (constant)	500
$\bar{C}$	[$]	Base consumption (subsistence level)	1000
C_W	[-]	Consumption fraction of wealth	0.05
C_π	[-]	Consumption fraction of net cash flow	0.1

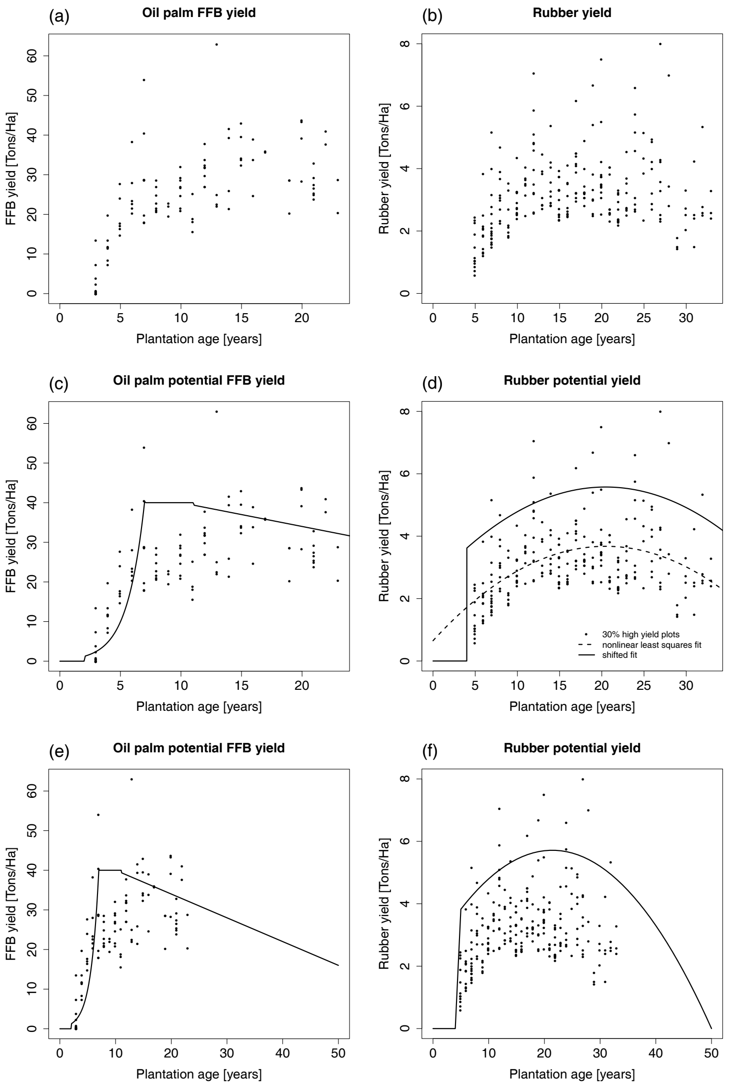

Figure S1.1: (a) Production of oil palm fresh fruit bunches [T/Ha] of the 30% highest yielding fields per plantation age. (b) Rubber production [T/Ha] of the 30% highest yielding fields per plantation age. (c), (d) nonlinear least square fit to oil palm and rubber data, respectively, (dotted line) and upwards shifted fit (95% of data under the curve). (e), (f) Optimal yield functions applied in the model (parameter values were rounded).

and a segmented linear regression with one and two breakpoints (Fig. S1.2 (c)). An AIC comparison of the three fits resulted in the lowest AIC for the segmented linear regression with two breakpoints. We therefore apply this function in the model and set the optimal labor input for the first three years to zero. One critical aspect is the extrapolation of labor inputs beyond the age where data were available. Apparently one reason why oil palm plantations generally have a lifespan of $25 - 30$ years is that after that period, yields decrease and harvesting becomes very difficult as the trees reach a height in which the fruit bunches are difficult to harvest with the conventional pole method. Therefore, we assume a steep increase in labor costs when palms reach a height after which the conventional harvesting method with long sticks is not possible anymore (see also [9] p. 303 ff. and p. 318). As plantation cycles in our data end after about 25 years, we assume, that at this time, labor costs increase and result in plantations being unprofitable. We calibrate the amount of labor needed by assuming that at this point, the net cash flow is approximately zero, given optimal inputs and observed input and output prices.

The optimal labor input function is therefore

$$labor_{oilpalm}(x) = \begin{cases} 0 \text{ , if } x \leq 2 \\ l_{o4} \cdot x + l_{o7} \text{ , if } x > 2 \text{ and } x \leq l_{o1} \\ l_{o5} \cdot x + l_{o8} \text{ , if } x > l_{o1} \text{ and } x \leq l_{o2} \\ l_{o6} \cdot x + l_{o9} \text{ , if } x > l_{o2} \text{ and } x \leq l_{o3} \\ l_{o10} \text{ , if } x > l_{o3} \end{cases} \tag{S1.46}$$

with parameters shown in Table S1.2 (see Fig. S1.2 (e)).

For rubber, we tested a constant, linear and hump-shaped function (see Eq. S1.45), with the AIC suggesting the hump-shaped curve (Fig. S1.2 (d)). However, since there was no large difference between the fits and labor input in rubber plantations seems to be rather steady over the years (regular tapping, harvesting and weeding), we decided to choose the constant function for optimal labor input. Therefore the optimal labor input for rubber is

$$labor_{rubber}(x) = \begin{cases} 0 \text{ , if } x \leq 4 \\ l_{r1} \text{ , if } x > 4 \end{cases} \tag{S1.47}$$

with parameter in Table S1.2 (see also Fig. S1.2 (f)).

Optimal technical input

To estimate optimal technical input for both land uses, we use the data on technical inputs from the 30% highest yielding fields per plantation age (see Fig. S1.3 (a) and (b)). As for labor, technical input in the immature phase of the plantation are considered as part of the investment. The data on technical inputs refer to seedlings, plant and animal waste, soil amendments, fertilizer, herbicides, machinery and input and output transportation (measured in fuel). Except seedlings, quantities of inputs are generally measured in liters per hectare and are also accumulated for each best performing field. Seedlings are plausibly assumed to have a weight of 1 kilogram. The data on technical inputs are very scattered for both land uses. For oil palm, the data suggest an increase in technical inputs over time, while the inputs for rubber seem quite uniform. For both land-use types we tested a linear and a constant relationship.

The resulting fits are shown in Figure S1.3 (c,d). For oil palm, although the AIC comparison suggests the linear increase, we decide for the constant relationship as the linear fit results in unrealistically high technical input when extrapolated for old plantations. Also fertilizer recommendations for oil palm plantations typically suggest a two-level fertilization scheme and differentiate only between immature and mature plantation phase [10]. Figure S1.3 (e) shows the applied relationship for optimal technical inputs, where inputs for the first three years are set to zero. The optimal technical input function is therefore

$$tinput_{oilpalm}(x) = \begin{cases} 0 \text{ , if } x \leq 2 \\ t_{o1} \text{ , if } x > 2 \end{cases} \tag{S1.48}$$

with parameters shown in Table S1.2.

For rubber we compared a linear regression with constant technical inputs and decide for the constant function which is also suggested by AIC. The applied relationship for optimal technical inputs in rubber is therefore

$$tinput_{rubber}(x) = \begin{cases} 0 \text{ , if } x \leq 4 \\ t_{r1} \text{ , if } x > 4 \end{cases} \tag{S1.49}$$

with parameter in Table S1.2 (see also Fig. S1.3 (f)).

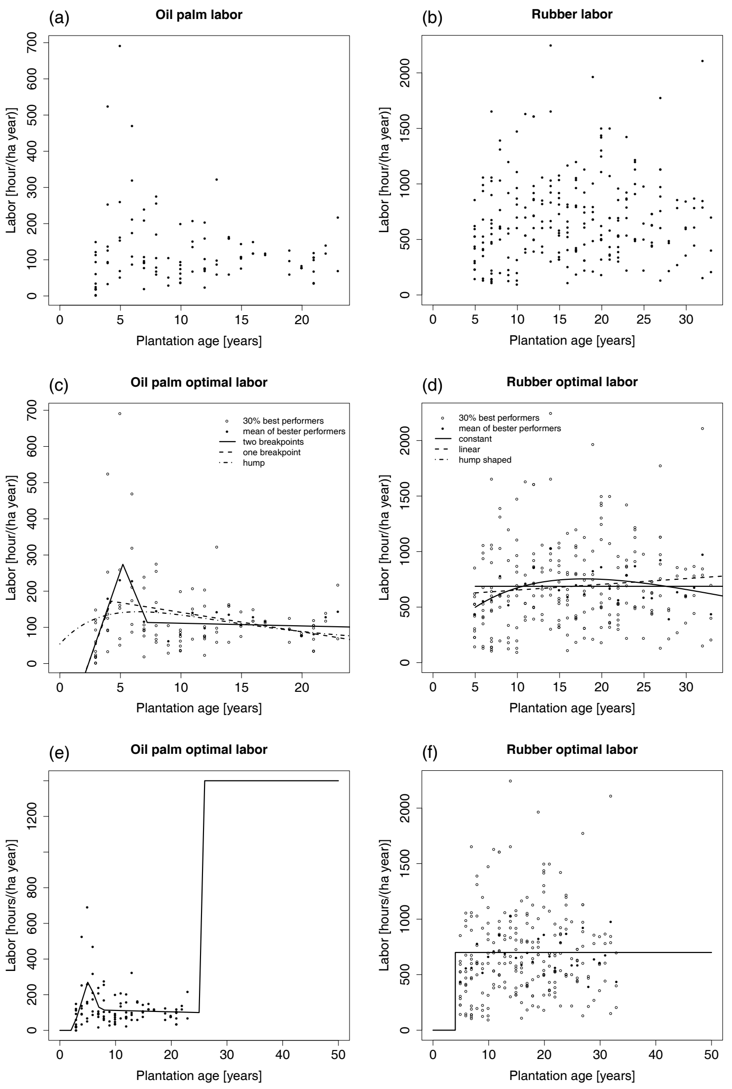

Figure S1.2: (a) Labor input for oil palm [h/(Ha year)] of the 30% highest production fields per plantation age. (b) Labor input for rubber [h/(Ha year)] of the 30% highest production fields per plantation age. (c) Different fits to the data: Segmented linear regressions with one and two breakpoints and a hump-shaped function of the form Equation S1.45. AIC results: 1257.5 (two breakpoints) < 1264.2 (one breakpoint) < 1271.5 (humped shape). (d) Different fits to the data: Constant labor input, linear regression and a hump-shaped function of the form Equation S1.45. AIC results: 3596.9 (hump shaped) < 3600.9 (linear) < 3601.4 (constant). (e), (f) optimal labor function applied in the model (parameter values were rounded).

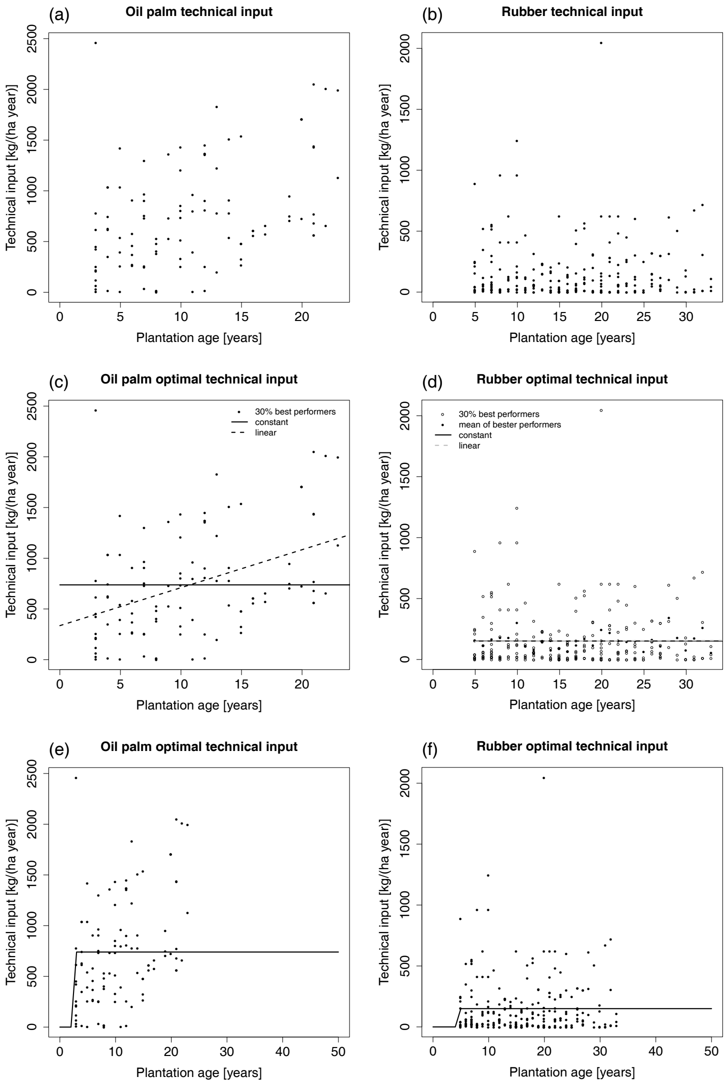

Figure S1.3: (a) Technical input in oil palm plantations [Kg/(Ha Year)] of the 30% highest production fields per plantation age. (b) Technical input in rubber plantations [Kg/(Ha Year)] of the 30% highest production fields per plantation age.(c) Different fits: exponential increase (continuous line), linear (dashed) and power law (dotted). AIC comparison: 1600.2 (exponential) < 1601.3 (linear) < 1602.9 (power law). (d) Different fits: constant function (continuous line) and linear regression (dashed). AIC comparison: 3358.7 (constant) < 3360.7 (linear). (e), (f) optimal technical input function applied in the model (parameter values were rounded).

Optimal capital input

The optimal capital input over time represents the capital stock of an oil palm or rubber plantation, i.e. the accumulated, discounted investment costs (see section *Production function, cash flows and capital accumulation*). During the immature period m of plantations we regard all labor costs and costs for technical inputs as investment costs. The accumulated value of costs for labor and technical inputs in this period are considered as total establishing costs of the plantation. All costs have been also derived from the household survey.

$$capstock_{oilpalm}(x) = \begin{cases} capstock_{oilpalm,x-1} * (1 + d_{o2}) + icost\_total_{o_{x,m}} & \text{, if } x \leq c_{o4} \\ capstock_{oilpalm,x-1} * (1 + d_{o3}) + icost\_total_{o_{x,m}} & \text{, if } x > c_{o4} \end{cases} \quad \text{(S1.50)}$$

$$capstock_{rubber}(x) = \begin{cases} capstock_{rubber,x-1} * (1 + d_{r2}) + icost\_total_{r_{x,m}} & \text{, if } x \leq c_{r4} \\ capstock_{rubber,x-1} * (1 + d_{r3}) + icost\_total_{r_{x,m}} & \text{, if } x > c_{r4} \end{cases} \quad \text{(S1.51)}$$

As investment costs for labor we include costs for the operations land clearing, pits taking, seedling transportation, planting and replanting, manure and fertilizer application, chemical and manual weeding, harvesting, pruning and marketing. Due to the high variance within the data on labor use, all labor costs per operation are calculated in multiplying the median hours of work per operation with the mean value of wages per operation. We also include costs for out-contracted labor. The costs for technical inputs are calculated in multiplying the idiosyncratic prices of inputs with the respective quantities of inputs. The respective inputs are seedlings, plant and animal waste, soil amendments, fertilizer, herbicides, machinery and input and output transportation. The resulting investment costs during the immature phase are shown in Figure S1.4 (a) and (b). As described in section *Production function, cash flows and capital accumulation*, we assume a positive depreciation rate, i.e. increasing capital stocks in young plantations, and afterwards a negative depreciation rate, i.e. decreasing capital stocks. All parameters concerning capital costs in oil palm and rubber plantations are given in Table S1.2. The resulting optimal capital inputs for the Leontief production function are shown in Figure S1.4 (c) and (d).

Optimal land input

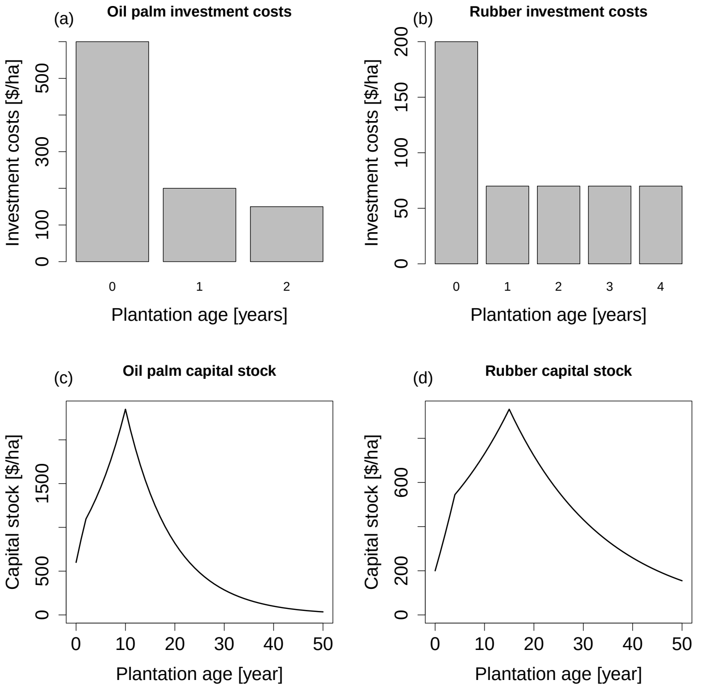

Figure S1.4: (a) Investment costs [$/Ha] for the first three years of an oil palm plantation. (b) Investment costs [$/Ha] for the first five years of a rubber plantation. As there was no large difference between the years 1 and 4, we apply the average of these years. (c), (d) Capital stocks over time.

Since we always calculate the Leontief production function based on a cell, the input for land is fixed to the cell size, in this case to 0.25 ha.

4.2.2 Costs, revenue & Cash flow

For the calculation of the different costs occurring in plantation agriculture over time, we use the household data to derive mean values for wages, prices of technical inputs and prices of land. We also include a price for capital, which captures the opportunity costs of capital referring to a rental rate of capital. Prices of fresh oil palm fruit bunches and rubber are also derived from the household survey (see Table S1.2).

All data are calculated as mean values over all fields considering only the mature period after the first three or five years for oil palm and rubber,

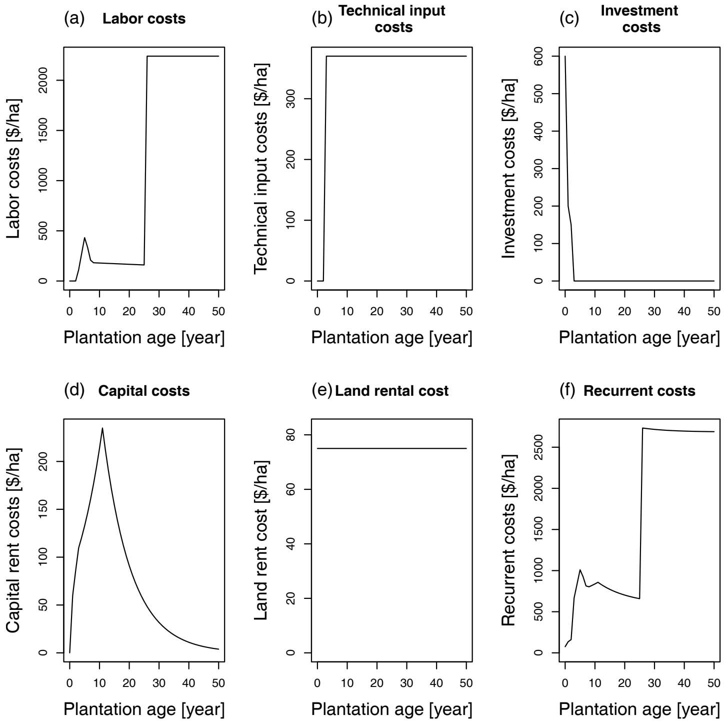

Figure S1.5: Overview of the different cost functions for oil palm over plantation age under optimal production inputs. Recurrent costs are the sum of labor, technical input, capital and land rental costs.

respectively. To receive the final mean value for wage measured in hours, we first calculate the average wage per day (per operation), which is divided by the average numbers of working hours (per operation). The kinds of operation we considered are land clearing, pits taking, seedling transport, replanting, manure and fertilizer application, chemical and manual weeding, harvesting, cutting leaves, marketing, intercultural operations and irrigation. From all mean wages per operations we took a final mean. For calculating the overall mean price of technical inputs, we consider only the most applied and widely representative technical inputs used in the survey, which are fertilizer and herbicides. For each input the mean price and quantity is calculated. To generate a final price and quantity, we weight the final quantities of fertilizer and herbicides with the respective mean price and divide them by the sum of both quantities.

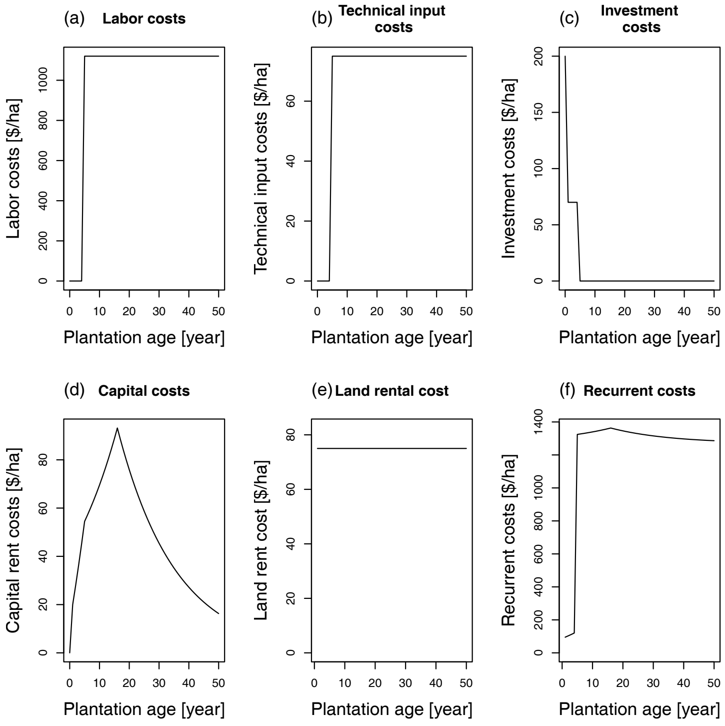

Figure S1.6: Overview of the different cost functions for rubber over plantation age under optimal production inputs. Recurrent costs are the sum of labor, technical input, capital and land rental costs.

The rental rates for capital (r_K) and land (r_l) (see Table S1.2) are calculated as the average interest rate for informal and formal credits reported in the household survey. The price for land (p_l) captures the average price for land per hectare, which has been sold between 2009 to 2012 (see Table S1.2). Applying theses factors to the optimal factor inputs derived in section *Production functions for oil palm and rubber*, we arrive at costs over the plantation lifetime presented in Figure S1.5 (oil palm) and Figure S1.6 (rubber).

Applying the average farm-gate prices as an example, we arrive at revenues and net cash flows shown in Figure S1.7 (a,b) and Figure S1.8 (a,b). Finally, Figure S1.7 (c,d,e) and Figure S1.8 (c,d,e) depict expected cash flows over the plantation lifetime (curves), as well as the expected cash flow for newly established plantations (straight lines). These expected cash flows are used

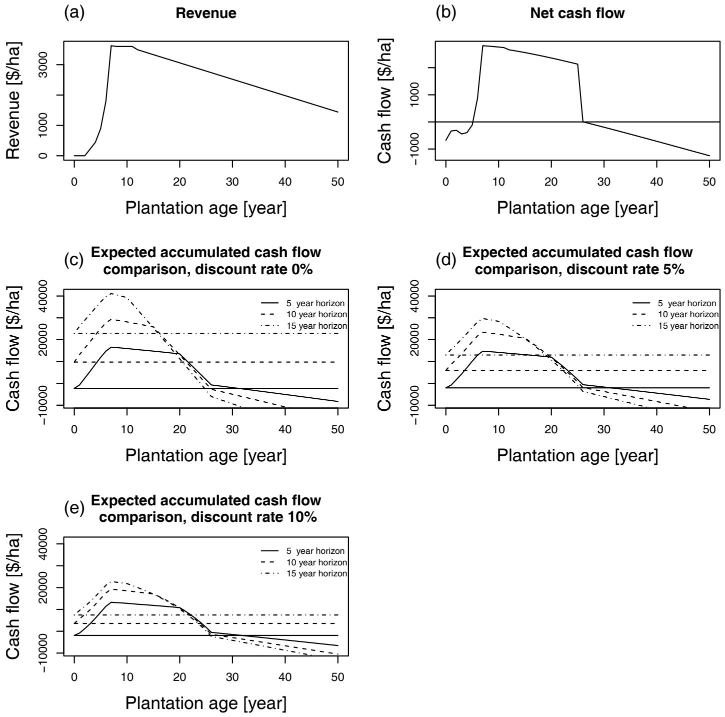

Figure S1.7: (a, b) Annual revenue and net cash flow of an oil palm plantation under optimal production inputs. (c), (d) and (e) Comparison of expected net cash flow of existing plantations (curves) with expected net cash flows from a newly established plantation (straight lines) under different planning horizons (5,10,15 years). Different fields represent different levels of discount rates (0, 0.05 and 0.1, respectively). The second intersection of each pair of lines marks the plantation age, in which replanting becomes the more profitable option.

in the model to compare different land-use change options (see section *Decision on land-use change and production*).

The accumulated expected net cash flow for newly established plantations over different time horizons and different price scenarios is shown in Figure S1.9. With the applied prices for oil palm fresh fruit bunches and rubber, rubber is the more profitable option, independent of the time horizon considered (Fig. S1.9 (a)). However, if the price relation between oil palm and rubber changes, e.g. with considerably lower prices for rubber, the

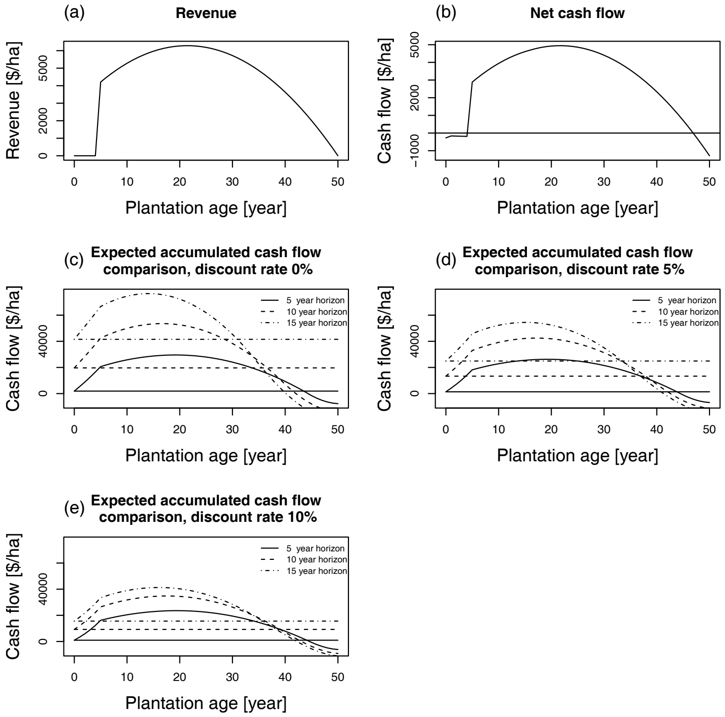

Figure S1.8: (a, b) Annual revenue and net cash flow of a rubber plantation under optimal production inputs. (c), (d) and (e) Comparison of expected net cash flow of existing plantations (curves) with expected net cash flows from a newly established plantation (straight lines) under different planning horizons (5,10,15 years). Different fields represent different levels of discount rates (0, 0.05 and 0.1, respectively). The second intersection of each pair of lines marks the plantation age, in which replanting becomes the more profitable option.

profitability can depend on the considered time horizon (Fig. S1.9 (b)).

4.2.3 Initial household inefficiency distribution

Yield data from the household survey were used to initialize the crop-specific inefficiencies of all households in the model (Fig. S1.1). The unrealized fractions of the optimal production functions (yield gaps) of each household and crop-type were calculated. In order to distribute these inefficiencies to the model households, different distribution functions were fitted against these inefficiency distributions of household survey data. The histogram of result-

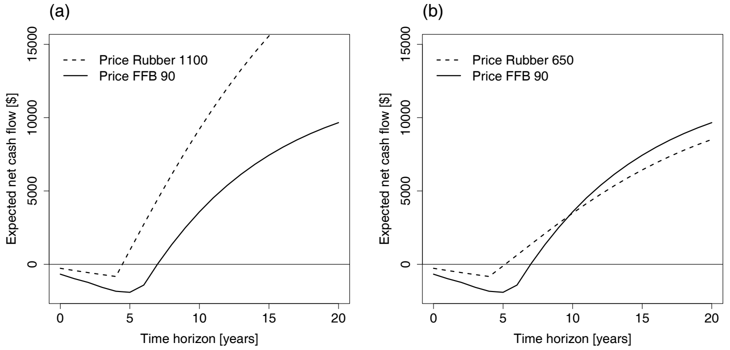

Figure S1.9: Accumulated expected net cash flows with discount rate of 0.1 for newly established oil palm and rubber plantations under different time horizons. (a) Prices for fresh fruit bunches FFB and rubber as derived from household data. (b) Hypothetical lower price for rubber.

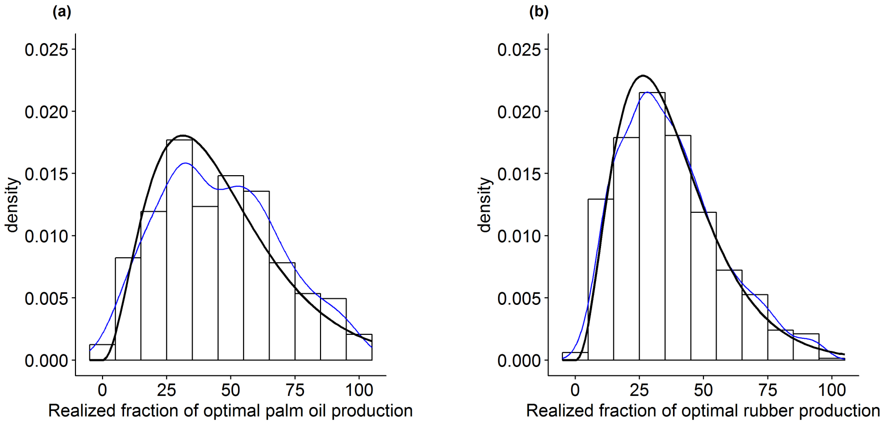

Figure S1.10: Oilpalm (a) and Rubber (b) efficiency distribution of survey households, calculated as realized fraction of optimal production. Bars show densities of efficiency classes, the blue line shows the density function, the black line shows the gamma distribution fit used for model initialization. The gamma distribution fit parameters are $\alpha = 3.168$ and $\lambda = 0.069$ for oilpalm efficiencies (a) and $\alpha = 3.445$ and $\lambda = 0.093$ for rubber efficiencies (b).

ing inefficiencies of households was slightly left-skewed, which resulted in a bad fit for the normal distribution. The issue was resolved by inverting the

inefficiencies to efficiencies $(1 - \delta_{i,l})$ which resulted in a right-skewed distribution, and performing a gamma distribution fit (see Fig. S1.10). These gamma distributions were then implemented in the model to distribute initial inefficiencies for oil palm and rubber by drawing random numbers from the corresponding distribution and inverting these values back to inefficiencies. Because the initial model households do already own differing numbers of oil palm and rubber fields, we additionally coupled the distribution of these two crop inefficiencies to prevent households from heaving a higher inefficiency for the crop type they currently cultivate at a larger proportion. To this end, during model initialization, each model household draws one inefficiency value from each gamma distribution. These values are only taken, if the crop type of the distribution with the lower inefficiency corresponds to the crop type the households cultivates at a larger proportion. Otherwise, a new pair of inefficiencies is drawn until these requirements are fulfilled. Households with equal proportions of both crop types draw inefficiencies from the gamma distribution without the need to fulfill any of these requirements.

4.3 Price dynamics

All farmers are assumed to receive the same price for the same crop. These prices are related to world market prices of the respective crops, but additionally we used information on price transmission from survey data. Farm-gate prices are considerably lower than world market prices mainly because of trade and transport margins. Average farm gate prices received by smallholders were 885 IDR/kg (about USD 0.09) of fresh fruit bunches for oil palm and 10412 IDR/kg (about USD 1.10) for rubber in the final quarter of 2012 (with an exchange rate of 9500 IDR/USD) see [1]. The world market price for rubber at that time was about 3.20 USD/kg; in April 2015 it had declined to 1.71 USD/kg. For palm oil, the prices of which cannot be readily compared to the price for fresh fruit bunches, prices also declined, but the decline was less pronounced; from 768 USD/metric ton in 10/2012 to 592 USD/metric ton in 04/2015 (all international price data from the World Bank).

Different options for price dynamics are implemented in the model and can be chosen from the GUI. Prices can be kept constant, or variable around the initial prices with a specifiable range of variation ("price-fluctuation-%"). In the latter case, the annual price variation is drawn from a uniform distribution. Prices can also be chosen as correlated, again with a specifiable

variation. In this case the price for the next year is calculated based on the current price with the variation again drawn from a uniform distribution. Fourth, prices can be chosen to follow a Gaussian random walk with crop-specific mean and standard deviation. For example, if p_n is the price per harvested ton fresh fruit bunches in year n, the price for the following year is determined as

$$p_{n+1} = p_n + r(\mu, \sigma) \qquad \text{(S1.52)}$$

where r is a normally distributed random variable with mean μ and standard deviation σ. While μ determines the expected slope of the price function, σ determines price volatility. Finally, prices can be set to follow trends based on nominal annual prices in the World Bank Commodity Price Data, using "Palm oil" for oil palm produce and "Rubber, TSR20" for rubber produce. The prices are adjusted using a land-use-specific multiplication factor so that the prices for 2012 match the farm-gate prices actually observed (and also used in the constant price scenario; see Table S1.2 and Figure S1.11).

The first analyses in this paper focus on basic model dynamics, so we apply the constant price option. In the second set of analyses we are interested in simulating real trends, and hence we choose the historical trends option.

4.4 Ecological model

4.4.1 Carbon storage

For the calculation of carbon stored in the vegetation of oil palm plantations, we use a function of [11], that estimates aboveground biomass (AGB) of oil palm plantations as a function of plantation age

$$AGB_{oilpalm}(age)[Mg\ ha^{-1}] = 18.95 * age^{0.5} \qquad \text{(S1.53)}$$

Assuming a carbon content of 41.3% and a constant root-shoot ratio of 0.35, i.e. 74% of total carbon is aboveground and 26% is below ground [12], we arrive at a vegetative carbon stock of

$$carbon_{oilpalm}(age)[Mg\ ha^{-1}] = (18.95 * age^{0.5} \cdot 0.413) \cdot 1.35 \qquad \text{(S1.54)}$$

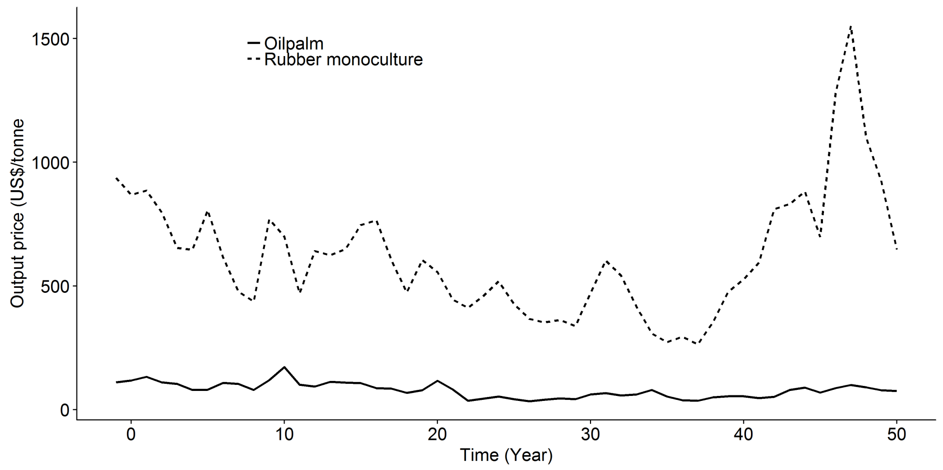

Figure S1.11: Prices for rubber and oil palm for each simulation year when running the model with historical price trends. The values for years 0-50 are adjusted from World Bank data for the corresponding years in the range 1963-2013.

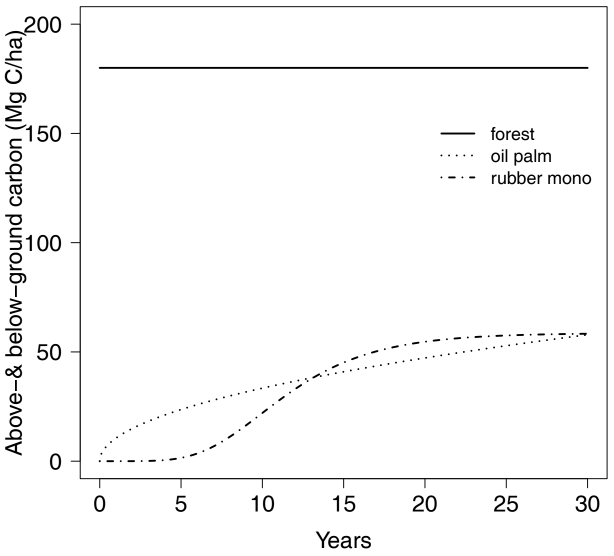

Figure S1.12: Carbon stocks of different land-use types

For rubber monoculture we apply the function for rubber trees in the Mato Grosso, Brazil from [13]

$$carbon_{rubbermono}(age)[Mg\ ha^{-1}] = 58.609 * exp(-13.696 * exp(-0.264 * age)) \tag{S1.55}$$

For forest we assign a constant carbon content (we do not consider the option of converting plantations into forest yet). A mean carbon stock value is derived from estimations of total biomass from plot data [14], applying a carbon content of 0.47% (default value for insular Asian tropical rainforests [15].

$$carbon_{forest}[Mg\ ha^{-1}] = 389 \cdot 0.47 \approx 180 \tag{S1.56}$$

The resulting carbon stocks are shown in Figure S1.12.

Appendix B. EFForTS-LGraf: Landscape generator

The landscape generator EFForTS-LGraf is an extended version of the simple process-based landscape generator G-RaFFe [16], which originally simulates the extension of fields from roads and creates binary maps with forest- and non-forest cells. For our purpose we added different land uses and households as an intermediate level between fields and landscape. Households can own several fields of different sizes with different land uses. Household locations are always close to roads. For the creation of maps for model initialization, we used a section of a real road map from the Jambi region. Main input parameters for the landscape generator are the density of farming households, the distribution of household sizes, the distribution of field sizes, and the fraction of the different land uses. For a full description of EFForTS-LGraf please contact jsaleck@gwdg.de.

1 Parametrization of EFForTS-LGraf

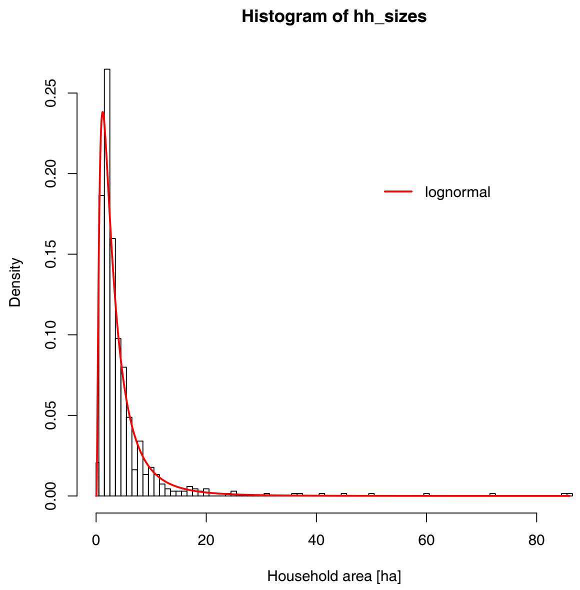

Figure S1.13: Histogram of household sizes with maximum likelihood fit of the log-normal distribution.

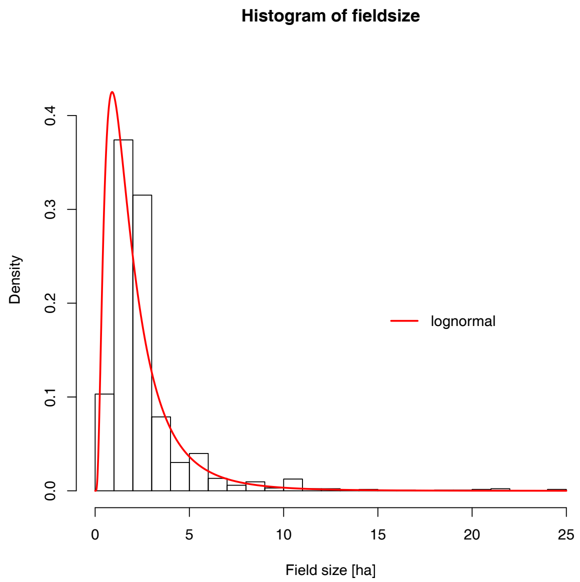

Figure S1.14: Histogram of field sizes with maximum likelihood fit of the log-normal distribution.

Household sizes

We use data from a household survey (701 households, [3, 4]) to determine the distribution of household sizes (= total area available for agricultural use). We scaled the histogram of household sizes to $[0, 1]$ and fitted the density functions of a log-normal distribution to the data (see Fig. S1.13) using maximum likelihood fitting (function *fitdistr* of the package MASS in the statistics software R). The resulting parameters for mean and standard deviation of household area are presented in Table S1.1, *S1 Appendix A*. Within the landscape generator, household sizes are determined by drawing a random number from the log-normal density function and rounding for the cell resolution (0.25 ha).

Field sizes

In the same manner as for household sizes, we use data from a household survey to determine the distribution of field sizes. We again fitted a log-normal distribution to the data (see Fig. S1.14). The resulting parameters for mean and standard deviation of field sizes are presented in Table S1.1, *S1 Appendix A*.

Appendix C. Initial household wealth

For the estimation of initial household wealth we use data on assets purchased by households between 2000 and 2012 from the household survey. Asset categories included for example cellphones, television, satellite dishes, motorbikes and cars, fridges and washing machines. Figure S1.15 shows the histogram of the cumulative value of purchased assets to which we fitted a log-normal distribution. We use this distribution as a proxy for household wealth. Since these purchased assets represent only a fraction of household wealth, we multiply the drawn values with a scaling factor (in this case 10), to obtain the initial values for household wealth.

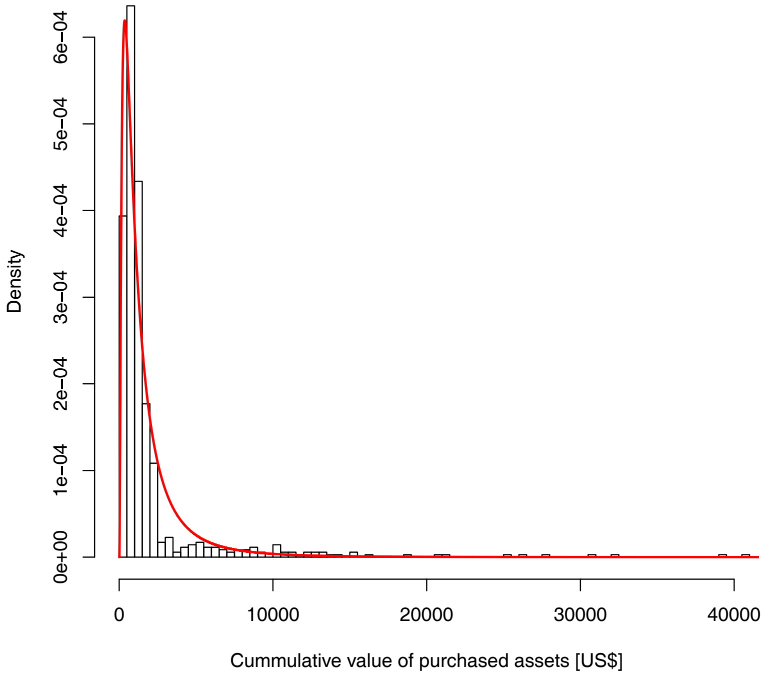

Figure S1.15: Histogram of cumulative value of purchased assets by households between 2000 and 2012.

Appendix D. Conversion of plantation plots under different land uses

Field studies show that a moderate proportion of oil palm was converted to other crops in the study area, while for rubber the respective conversion was much lower (overall, 14.47% vs. 0.99%; see Table S1.4). The low proportion of rubber suggests that conversion of fields of rubber plantations to oil palm does not generally occur. Evidence from the field also indicates that during times of low rubber output prices, fields are left to fallow until prices are profitable again (V. Krisha, personal communication).

Table S1.4: Share of plantation plots under different land uses at the time of acquisition. [a]Includes land transformations and transactions before 1993 also. ***, **, *: The difference in the proportions of oil palm and village plots developed from a given type of land use is statistically significant at 0.10, 0.05 and 0.10 levels, respectively. Source: Farm-household survey data (2013).

	Overall[a]		1993-2002		2003-2012	
	Oil palm	Rubber	Oil palm	Rubber	Oil palm	Rubber
No conversion	19.12	35.22***	25.60	40.52***	18.25	46.56***
Other crops	**14.47**	0.99***	10.71	1.04***	14.29	1.62***
Primary forest	13.95	32.54***	10.12	26.75***	13.49	19.84*
Secondary forest (bush and grass lands)	33.07	27.38**	28.57	29.61	47.62	31.58***
No idea	19.38	3.87***	25.00	2.08***	6.35	0.40***
Overall	100.00	100.00	100.00	100.00	100.00	100.00
Number of plots	*387*	*1008*	*168*	*385*	*126*	*247*

Appendix E. Additional model results

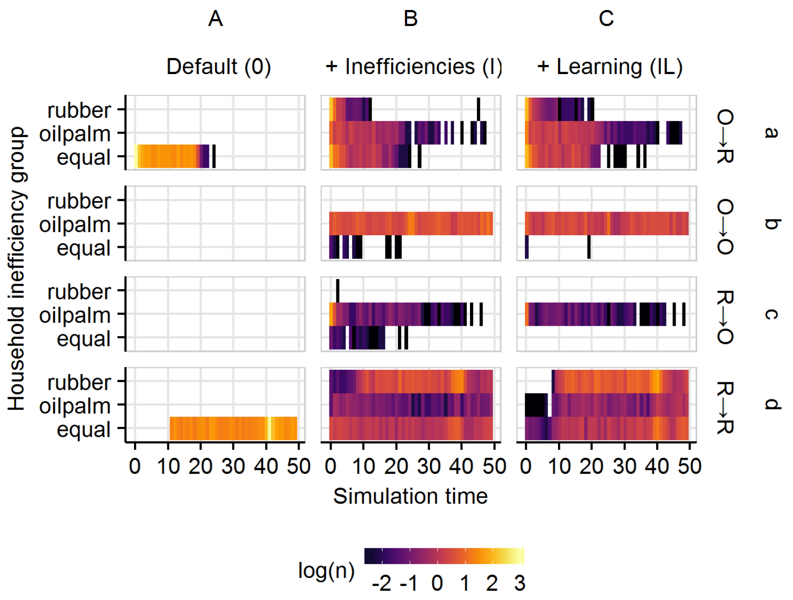

Figure S1.16: Land-use change and replanting of fields under constant prices scenario for agents with no inefficiencies and no learning (A), heterogeneous inefficiencies and no learning (B) and heterogeneous inefficiencies and learning (C). Households have been segregated into three classes (y-axis) based on the difference in crop inefficiencies. Households with less then 10% difference between rubber and oilpalm inefficiency are classified as *equal*. Households with at least 10% lower rubber inefficiency than oilpalm inefficiency are classified as *rubber experts*. Households with at least 10% lower oilpalm inefficiency than rubber inefficiency are classified as *oilpalm experts*. The four columns show the four possible land-use change decisions that reset field age to zero: Land-use change from oilpalm to rubber (a), replanting of oilpalm fields (b), land-use change from rubber to oilpalm (c) and replanting of rubber fields (d). The colored strips show for each time step and household class the logarithmic mean number of fields (derived from 20 model replications) with land-use change of the respective land-use-change option.

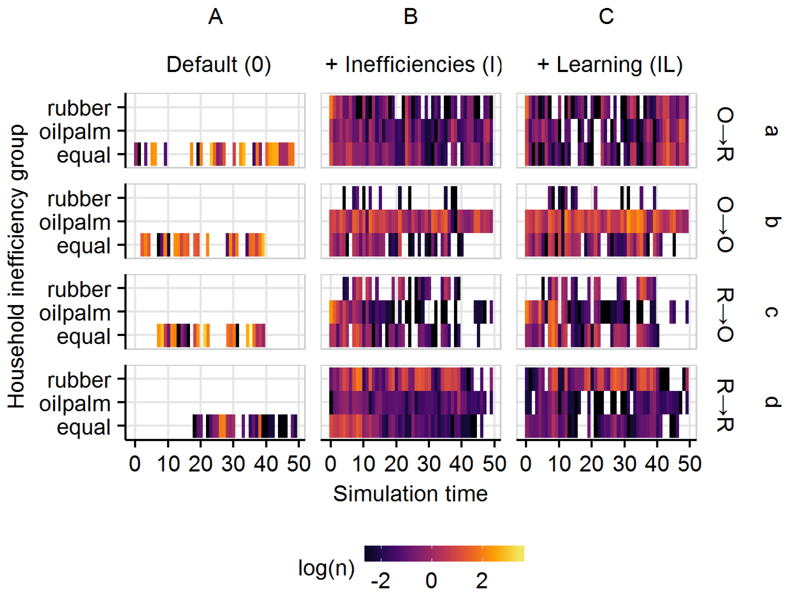

Figure S1.17: Land-use change and replanting of fields under historical price trends scenario for agents with no inefficiencies and no learning (A), heterogeneous inefficiencies and no learning (B) and heterogeneous inefficiencies and learning (C). Households have been segregated into three classes (y-axis) based on the difference in crop inefficiencies. Households with less then 10% difference between rubber and oilpalm inefficiency are classified as *equal*. Households with at least 10% lower rubber inefficiency than oilpalm inefficiency are classified as *rubber experts*. Households with at least 10% lower oilpalm inefficiency than rubber inefficiency are classified as *oilpalm experts*. The four columns show the four possible land-use change decisions that reset field age to zero: Land-use change from oilpalm to rubber (a), replanting of oilpalm fields (b), land-use change from rubber to oilpalm (c) and replanting of rubber fields (d). The colored strips show for each time step and household class the logarithmic mean number of fields (derived from 20 model replications) with land-use change of the respective land-use-change option.

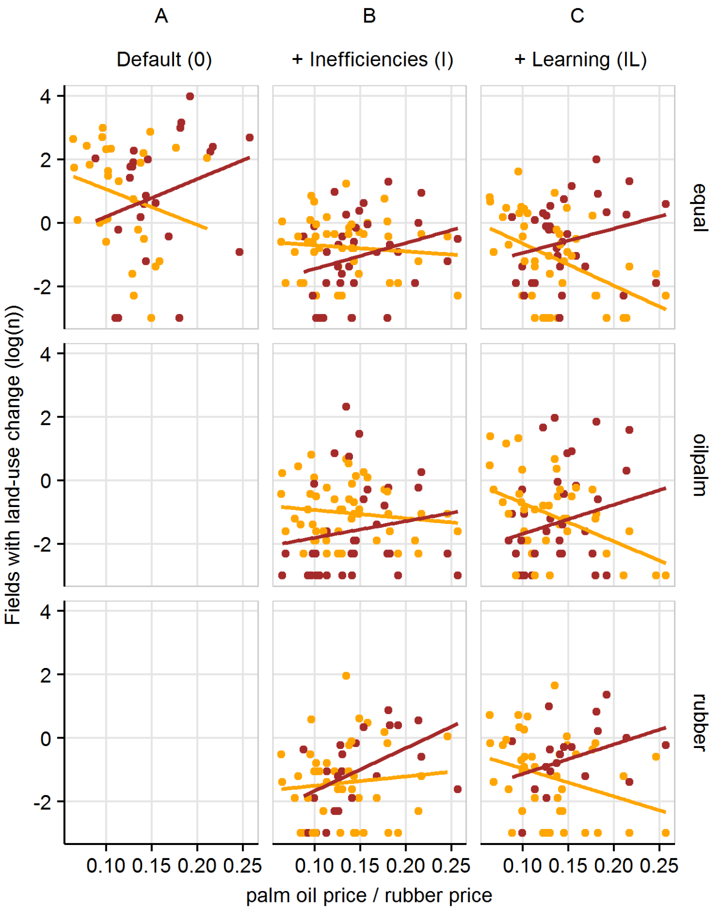

Figure S1.18: Correlation of land-use change and output prices under historical price trends scenario for agents with no inefficiencies and no learning (A), heterogeneous inefficiencies and no learning (B) and heterogeneous inefficiencies and learning (C). Households have been segregated into three classes based on the difference in crop inefficiencies. Households with less then 10% difference between rubber and oilpalm inefficiency are classified as *equal*. Households with at least 10% lower rubber inefficiency than oilpalm inefficiency are classified as *rubber experts*. Households with at least 10% lower oilpalm inefficiency than rubber inefficiency are classified as *oilpalm experts*. The colored points refer to the logarithmic mean number of fields (derived from 20 model replications) with land-use change from oilpalm to rubber (dark red points) and from rubber to oilpalm (dark yellow points) in dependence of the current price ratio (palm oil price / rubber price). The linear models show the general trend for each land-use change option.

References

1. Euler M, Schwarze S, Siregar H, Qaim M. Oil Palm Expansion among Smallholder Farmers in Sumatra, Indonesia. Journal of Agricultural Economics. 2016;67(3):658–676. doi:10.1111/1477-9552.12163.

2. Diewert WE. An Application of the Shephard Duality Theorem: A Generalized Leontief Production Function. Journal of Political Economy. 1971;79(3):481–507.

3. Euler M, Krishna V, Fathoni Z, Hermanto S, Schwarze S, Qaim M. Ecological and Socioeconomic Functions of Tropical Lowland Rainforest Transformation Systems (Sumatra, Indonesia), Household Survey 2012. Göttingen, Germany; Jambi, Indonesia; Bogor, Indonesia. 2012;Georg-August University of Goettingen, Bogor Agricultural University, and University of Jambi (datasets).

4. Faust H, Schwarze S, Beckert B, Brümmer B, Dittrich C, Euler M, et al. Assessment of socio-economic functions of tropical lowland transformation systems in Indonesia - sampling framework and methodological approach. EFForTS Discussion Paper Series. 2013;(1):University of Goettingen.

5. Krishna VV, Pascual U, Qaim M. Do emerging land markets promote forestland appropriation? Evidence from Indonesia. EFForTS Discussion Paper Series. 2014;(7):University of Goettingen.

6. Euler M, Krishna V, Schwarze S, Siregar H, Qaim M. Oil Palm Adoption, Household Welfare, and Nutrition Among Smallholder Farmers in Indonesia. World Development. 2017;93:219–235. doi:10.1016/j.worlddev.2016.12.019.

7. Krishna VV, Euler M, Siregar H, Fathoni Z, Qaim M. Farmer heterogeneity and differential livelihood impacts of oil palm expansion among smallholders in Sumatra, Indonesia. EFForTS Discussion Paper Series. 2015;(13):University of Goettingen.

8. Hoffmann MP, Castaneda Vera A, van Wijk MT, Giller KE, Oberthür T, Donough C, et al. Simulating potential growth and yield of oil palm (Elaeis guineensis) with PALMSIM: Model description, evaluation and application. Agricultural Systems. 2014;131:1–10. doi:10.1016/j.agsy.2014.07.006.

9. Corley RHV, Tinker PBH. The Oil Palm. John Wiley & Sons; 2008.

10. Comte I, Colin F, Whalen JK, Grunberger O, Caliman JP. Chapter three - Agricultural Practices in Oil Palm Plantations and Their Impact on Hydrological Changes, Nutrient Fluxes and Water Quality in Indonesia: A Review. Advances in Agronomy. 2012;116:71–124. doi:http://dx.doi.org/10.1016/B978-0-12-394277-7.00003-8.

11. Germer J, Sauerborn J. Estimation of the impact of oil palm plantation establishment on greenhouse gas balance. Environment, Development and Sustainability. 2008;10(6):697–716. doi:10.1007/s10668-006-9080-1.

12. Syahrinudin. The potential of oil palm and forest plantations for carbon sequestration on degraded land in Indonesia. Ecology and Development Series. 2005;28:1–107.

13. Wauters JB, Coudert S, Grallien E, Jonard M, Ponette Q. Carbon stock in rubber tree plantations in Western Ghana and Mato Grosso (Brazil). Forest Ecology and Management. 2008;255(7):2347–2361. doi:10.1016/j.foreco.2007.12.038.

14. Kotowska MM, Leuschner C, Triadiati T, Meriem S, Hertel D. Quantifying above- and belowground biomass carbon loss with forest conversion in tropical lowlands of Sumatra (Indonesia). Global Change Biology. 2015;21(10):3620–3634. doi:10.1111/gcb.12979.

15. IPCC. 4: Forest Land. In: 2006 IPCC Guidelines for National Greenhouse Gas Inventories. vol. 4. IGES; 2006. p. 4.1–4.83.

16. Pe'er G, Zurita GA, Schober L, Bellocq MI, Strer M, Müller M, et al. Simple Process-Based Simulators for Generating Spatial Patterns of Habitat Loss and Fragmentation: A Review and Introduction to the G-RaFFe Model. PLOS ONE. 2013;8(5):e64968. doi:10.1371/journal.pone.0064968.
