# Supplementary figures and images for: Land-use change in oil palm dominated tropical landscapes—An agent-based model to explore ecological and socio-economic trade-offs
Source: PLoS One. 2018 Jan 19;13(1):e0190506. doi: 10.1371/journal.pone.0190506 (PMC5774713; doi:10.1371/journal.pone.0190506)

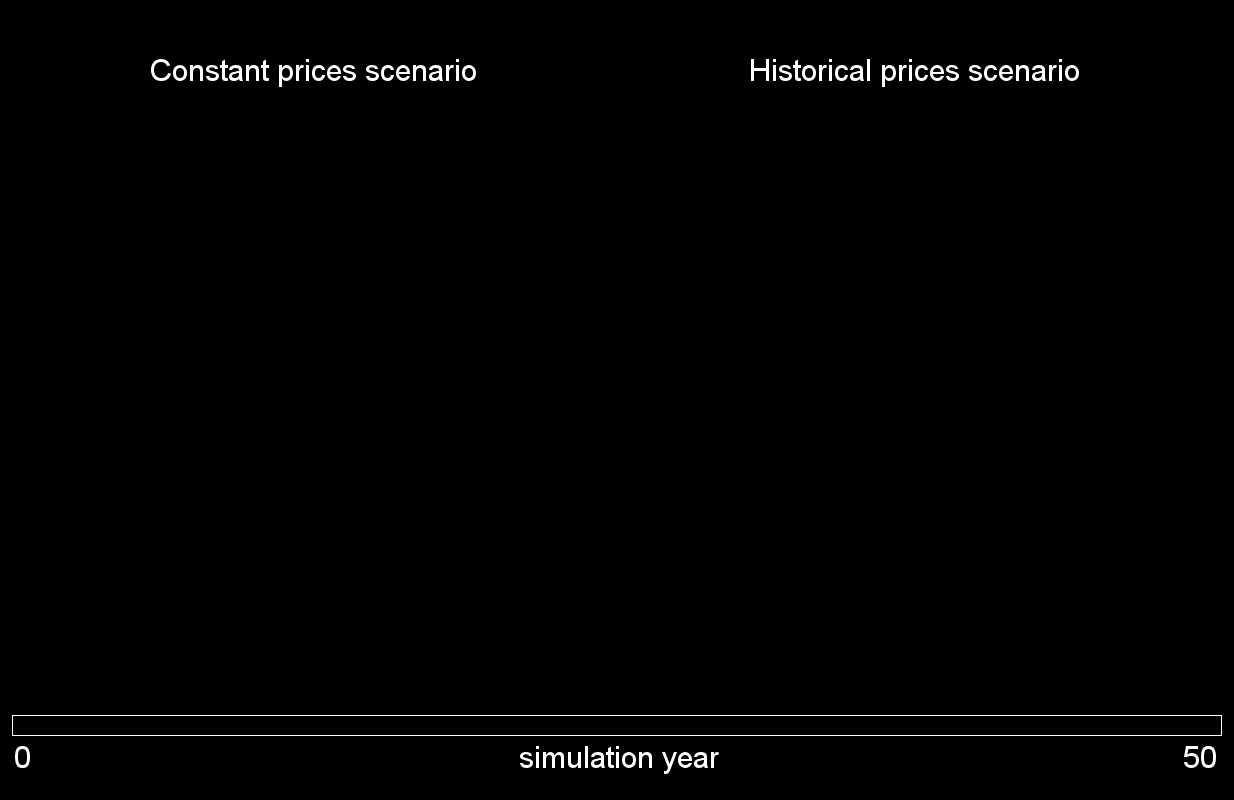

Supplement: S1 Fig — (GIF) [file pone.0190506.s002.gif]
